# Evolving understanding of autoimmune mechanisms and new therapeutic strategies of autoimmune disorders

**DOI:** 10.1038/s41392-024-01952-8

**Published:** 2024-10-04

**Authors:** Yi Song, Jian Li, Yuzhang Wu

**Affiliations:** 1 Institute of Immunology, PLA, Third Military Medical University (Army Medical University), Chongqing, China; 2 Chongqing International Institute for Immunology, Chongqing, China

**Keywords:** Immunological disorders, Immunotherapy

## Abstract

Autoimmune disorders are characterized by aberrant T cell and B cell reactivity to the body’s own components, resulting in tissue destruction and organ dysfunction. Autoimmune diseases affect a wide range of people in many parts of the world and have become one of the major concerns in public health. In recent years, there have been substantial progress in our understanding of the epidemiology, risk factors, pathogenesis and mechanisms of autoimmune diseases. Current approved therapeutic interventions for autoimmune diseases are mainly non-specific immunomodulators and may cause broad immunosuppression that leads to serious adverse effects. To overcome the limitations of immunosuppressive drugs in treating autoimmune diseases, precise and target-specific strategies are urgently needed. To date, significant advances have been made in our understanding of the mechanisms of immune tolerance, offering a new avenue for developing antigen-specific immunotherapies for autoimmune diseases. These antigen-specific approaches have shown great potential in various preclinical animal models and recently been evaluated in clinical trials. This review describes the common epidemiology, clinical manifestation and mechanisms of autoimmune diseases, with a focus on typical autoimmune diseases including multiple sclerosis, type 1 diabetes, rheumatoid arthritis, systemic lupus erythematosus, and sjögren’s syndrome. We discuss the current therapeutics developed in this field, highlight the recent advances in the use of nanomaterials and mRNA vaccine techniques to induce antigen-specific immune tolerance.

## Introduction

Autoimmune disorders such as multiple sclerosis (MS), type 1 diabetes (T1D) and rheumatoid arthritis (RA) occur when autoreactive immune cells, especially T cells and B cells are overactivated and recruited to cause self-tissue damage.^1,2^ By far, researchers have discovered about 150 types of autoimmune diseases and adopted a series of treatment measures.^3^ The diversity and rapid rise of autoimmune diseases challenge the health care system and the entire pharmaceutical industry. Current drugs available for the treatment of autoimmune diseases are non-specific and have side-effects such as infection, allergy and malignant disease.^4^ Instead, antigen-specific immunotherapies for autoimmune diseases aim to induce tolerization toward autoantigens without suppressing the systemic immunity.

New therapies are developed based on a detailed understanding of the mechanisms of autoimmune diseases.^4^ In this review, we describe the epidemiology, clinical diagnosis, pathogenesis, mechanisms and therapies of autoimmune diseases. We provide a timeline to summarize the significant advances in the field of antigen-specific immunotherapy for the treatment of autoimmune diseases. We describe the different strategies developed for non-specific biotherapeutics as well as antigen-specific immunotherapy, and the delivery methods to induce immune tolerance. We also summarize the Food and Drug Administration (FDA) approved drugs for autoimmune diseases and antigen-specific therapies that have entered clinical trials. The most recent biomaterial-based and mRNA vaccine strategies for inducing antigen-specific tolerance are highlighted.

## Basic information of autoimmune diseases

### Common epidemiology

Autoimmune diseases have been shown to affect 3–5% of the population and become one of the most important public health problems.^5,6^ Recently, Conrad et al. reported a population-based cohort study of 19 autoimmune diseases in the UK about 22,009,375 individuals from 2000 to 2019.^7^ During this period, 978,872 individuals were newly diagnosed with autoimmune diseases and the average age of these individuals was 54, however, autoimmune diseases can occur in almost all age groups (0–95 years). Besides, 63.9% of these newly diagnosed patients are female, and the age and sex standardized incidence rates increased. The incidence of celiac disease and Sjogren’s syndrome increased. Autoimmune diseases affect about 10% of the population in this study and consume considerable social resources.^7^ In addition, some autoimmune diseases show seasonal and regional variations which may provide a guidance direction for autoimmune disease prevention and therapy.^8,9^

### Immune dysregulation

**Fig. 1:**
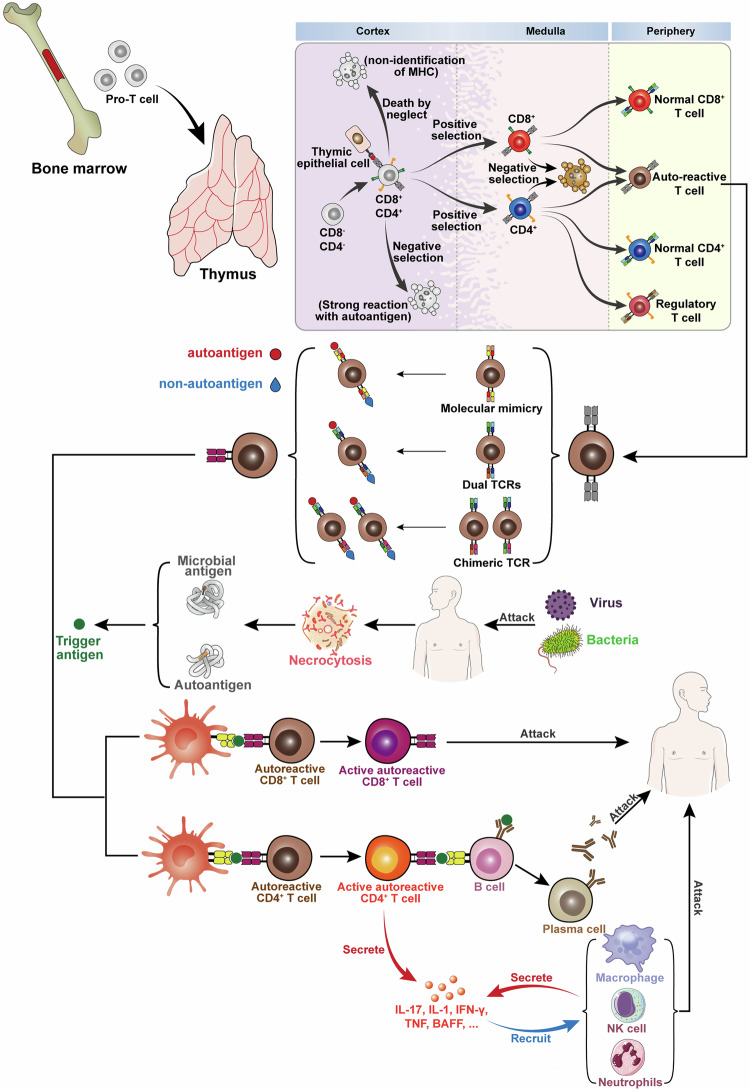
Pattern diagram of the mechanisms of autoimmune diseases. After differentiation of hematopoietic stem cells, progenitor T cell (pro-T cell) will leave the bone marrow and enter the thymus, and differentiate from double-negative (DN) T cells into double-positive (DP) T cells. Under death by neglect, negative selection, and positive selection via thymic epithelial cells, single positive T cells with low avidity to autoantigens-MHC complexes survive and differentiate into CD4 or CD8 and enter the periphery. However, some autoreactive T cells can avoid these select clearance effects and enter the peripheral. These autoreactive T cells include three types: (1) molecular mimicry, TCR can recognize the autoantigens and foreign antigens similar to autoantigens such as viruses and some bacteria. (2) dual TCRs, one TCR can recognize the non-autoantigens and another can recognize the autoantigens. (3) chimeric TCR, different Vα and Vβ combinations can recognize the autoantigens and non-autoantigens. Viruses, bacteria, and other autoantigens lead to the necrosis of autologous cells and result in the release of autoantigens. Some bacteria similar to autoantigens can induce the activation of these T cells susceptible to autoantigens and promote the autoimmune disease. Besides, the stimulation of external antigens can promote the continuous inflammatory environment and lead to the highly activated immune state of T and B cells. These T cells can secrete various inflammatory cytokines, activate B cells and recruit many immune cells, and induce inflammatory reaction. Eventually this will lead to the occurrence and development of autoimmune diseases. (Part of the figure was modified from Servier Medical Art(http://smart.servier.com/), licensed under a Creative Common Attribution 4.0 Generic License. (https://creativecommons.org/licenses/by/4.0/)

Autoimmune diseases are characterized by immune disturbances that cause the aberrant activation of autoreactive immune cells, resulting in tissue damage. Immune tolerance is established both centrally and peripherally.^10,11^ As we all know, T cells undergo positive and negative selection in the thymus before entering the periphery to perform immune functions. The negative selection of autoreactive T cells in the thymus is the major mechanism of central immune tolerance^12^ (Fig. 1). Besides, peripheral tolerance-related mechanisms can further limit the expansion of autoreactive cells through clonal deletion, immune anergy or the induction of regulatory T cells.^13,14^ Peripheral clonal deletion is mainly through activation-induced cell death or restimulation-induced cell death (RICD).^15,16^ Immune anergy mainly exerts its mechanism through various costimulatory molecules (like CTLA-4) and immune regulatory-related cells.^17^ Besides, follicular DCs and helper T cells can also affect the immune tolerance condition.^18^

T cells and B cells have been well investigated for their role in initiating and sustaining of autoimmune diseases. During autoimmunity, autoreactive T cells infiltrated into the target tissue. CD8^+^ cytotoxic T cells can directly contact and kill the targeted cells. CD4^+^ T cells can release large amounts of proinflammatory factors or provide activation signals to B cells. These proinflammatory factors recruit many myeloid inflammatory cells to specific tissue and executive-related immune response. Mature B cells can differentiate into plasma cells and secrete a large number of autoantigen-targeting antibodies (Fig. 1). Autoantibodies activate the complement system or kill the targeting cells by antibody-dependent cell-mediated cytotoxicity. Besides, the formation of antigen-antibody complexes is critical for some autoimmune diseases such as SLE. In SLE, these complexes deposit in the kidney and stimulate the inflammatory response in local tissue to cause tissue damage.^19^

### Genetic factors

The breakdown of immune tolerance is based on genetic susceptibility. Human leukocyte antigens (HLA) gene fragment is the most relevant gene to immune system, and variation of some loci in this region may promote the occurrence of autoimmune diseases.^20^ PTPN22 gene outside the major histocompatibility complex (MHC) region plays an important role in many autoimmune diseases including RA, Systemic lupus erythematosus (SLE), etc.^21,22^ Besides, the variation of gene coding antigens can also promote the occurrence of autoimmune diseases.^23^ Although most autoimmune diseases are polygenetic, some monogenic variations also have a strong impact on autoimmune diseases such as complement-related genes, nuclease hydrolysis-related genes and immune regulation-related genes.^24–26^ Researchers also emphasize the epigenetic factors for autoimmune diseases.^27–29^ Females are more frequently affected by autoimmune diseases than males. This gender bias is associated with hormones and X chromosome.^30–32^

### Environmental triggers

Many environmental factors have been associated with the development of autoimmune diseases. Meanwhile, these factors also reflect the pathogenesis of autoimmune diseases. Molecular mimicry hypothesis suggests that molecular mimicry is one of the environmental factors that leads to the break of tolerance and elicits autoimmune responses. It occurs when exogenous antigens similar to autoantigens induce the activation of autoreactive T cells or B cells in a susceptible individual.^33^ In addition, the models of dual TCRs and chimeric TCR also raise other possibilities^34,35^ (Fig. 1). Researchers also considered the exposure of pathogen-associated molecular patterns such as endotoxin or lipopolysaccharide repeatedly can stimulate the innate immune responses and enhance the adaptive immune responses. T and B cells will be in a state of highly activated immune state in this case.^36^ Multiple infectious agents have been suggested to play a role in autoimmune diseases. For example, Epstein-Barr virus (EBV) can stimulate the innate and adaptive immune responses simultaneously because the protein structure of this virus is similar to RNA binding proteins.^37–39^ EBV has been associated with many autoimmune diseases such as MS,^40^ SLE^41^, and RA.^42^ The disturbance of the composition of microbiota (Fungi, bacteria, viruses, etc.) located in gut, mouth and skin of the host can affect the host immune system^43^ (Fig. 1). Besides, these coexisting microorganisms can translocate in blood circulation and locate in the tissue to trigger immune responses locally.^44,45^ Some microorganisms may regulate biological metabolic process to promote immunity.^46,47^ The nutrition change in some Western countries coincides with the rise in autoimmune diseases. This may be explained by interactions among dietary, gut microbiota, metabolites and immune cells.^48^ Smoking has also been reported to affect the progress of autoimmune diseases but the mechanism is still not clear.^49^

### Molecular signaling pathways related with autoimmune diseases

**Fig. 2:**
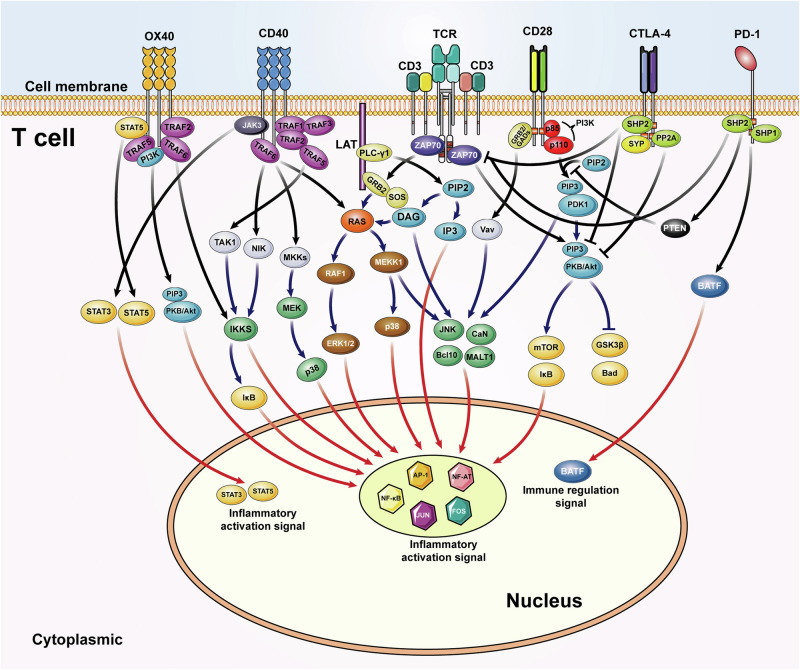
Related molecular pathways and membrane surface markers. OX40-OX40L, TRAF2/TRAF5/TRAF6 will induce the form of IKKα/β/γ which further leads to NF-κB entering the nucleus. Besides, OX40-OX40L can promote PI3K/Akt pathway and cause STAT5 to enter the nucleus. CD40-CD40L will recruit various downstream molecules. TRAF1, TRAF2, TRAF3, and TRAF5 bind competitively the one CD40 tail site and TRAF6 can bind to another individually. They can promote the Ras/ERK pathway and the non-classical NF-κB pathway, NIK pathway. Besides, it can promote the TAK1 and MKKs/p38 pathways. CD40-CD40L can start the JAK3/STATs pathway. CD28-B7-1/B7-2 also provides the activation signal. After the tyrosine phosphorylation of the YMNM fragment, the subunit p85 of PI3K binds to YMNM. PI3K will recruit PDK1 and PKB/Akt, and PKB can phosphorylate downstream targets such as mTOR, IκB, GSK3β and Bad after PKB is phosphorylated by PDK1 which leads to an increase of the transcriptional activity of NF-κB and NFAT. Besides, CD28 signal will recruit GRB2/GADs and increase NF-κB, NFAT, and AP1 by Vav catalysis. CTLA-4 also binds B7-1/B7-2, but it transmits the suppression signal to downstream. The specific process is through the inhibition of ZAP70 and PI3K/Akt pathway by recruitment of SHP2 and inhibition of PI3K/Akt pathway by PP2A. The combination with PD-1 and PD-L1 leads to the activation of the tyrosine phosphorylation of the ITIM and ITSM at the tail of PD1. SHP-1 or SHP-2 can bind the ITSM and promote the expression of PTEN which can further inhibit the activation of PI3K/Akt pathways and ZAP70. The SHP2 can also promote the BATF to enter the nucleus. It leads to the inhibition of T cell proliferation and inflammatory progression. This inhibitory process may be somewhat similar to the CTLA-4 pathway

The activation of immune cells requires the involvement of several molecular pathways and membrane surface molecules, which are closely related with autoimmune disease pathogenesis^50^ (Fig. 2). Here we also make a general description of some signal pathways and related molecules about T and B cell activation. CD28 system-related molecular pathways including CD28, CTLA4, and the shared ligands (CD80 and CD86) mainly are associated with the activation, proliferation and survival of T cells and this pathway is PI3K dependent. The YMNM sequence at the tail of CD28 is activated, and then the p85 subunit is combined with it subsequently. Activated PI3K will recruit proteins such as PDK1 and PKB/AKT, and then induce the activation of downstream targets, including mTOR, IκB, GSK3β and Bad, which can regulate the activity of transcription factors.^51,52^ CD28-deficient mice show the impaired germinal center and fail to generate normal levels of immunoglobulin.^53–55^ CD28 deficiency can delay disease progression and reduce disease severity in various autoimmune disease models including EAE,^56^ MRL/lpr model of SLE^57^ and collagen-induced arthritis model of RA.^58^ CTLA4 pathway can inhibit the CD28 pathway by binding the same ligands (CD80 and CD86).^59–61^ Targeting CTLA4 drugs have been applied in clinical trials in psoriasis and juvenile idiopathic arthritis.^62,63^ ICOS pathway will be upregulated after activation of CD4^+^ T cells and it can also mediate PI3K-AKT signal pathway for cell activation.^64,65^ ICOS is closely related to T follicular helper (Tfh) cells via IL-21 and IL-4 secretion.^66^ Hence, autoantibodies-related autoimmune diseases mentioned above are greatly influenced by ICOS pathway.^67–69^ Other CD28 superfamily members also include PD1 and BTLA which can inhibit immune activation.^70,71^ PD1 agonists can effectively reduce the severity of collagen-induced arthritis^72,73^ and colitis models induced by dextran sodium sulfate or T cell transfer.^74^

The binding of CD40 on T cells and CD40L on B cells can promote B cells interior recruits the TNFR-associated factors (TRAFs), and reaction molecules include NIK, inhibitor of NF-κB kinase and TPL2 which lead to the activation of transcription factors such as NF-κB and AP1 at last.^75^ CD40-CD40L is a universal signal for various immune cells to induce widespread downstream immune function, especially in humoral immunity^76^ including T cell-dependent antibody production, formation of germinal centers and differentiation of memory B cells.^77,78^ In addition to antibody induction, CD40 pathway can also result in inflammatory factors including TNF and matrix metalloproteinases (MMPs) for joint destruction in RA.^79^ CD40 is also continuously expressed on salivary gland ductal epithelial cells and endothelial cells^80^ to up-regulate adhesion molecules for inflammatory progression in Sjögren’s syndrome (SS).^81,82^ Blocking CD40 pathway can decrease disease activity and clinical remission in a RA clinical trial.^83^ The similar therapeutic effect also appears in SS model treated with anti-CD40L.^84^ Besides, the binding of OX40 on T cells and OX40L on antigen presenting cells (APCs) is another important pathway for immune activation. The downstream of OX40 will induce many signal pathways such as PI3K-AKT, NF-κB, and MAPK by recruiting TRAF2, TRAF5, and other molecules.^85,86^ TNF receptor family also includes TNF, BAFF, APRIL, RANK, etc.^50^ OX40-OX40L mainly promotes the differentiation of helper T cell subsets, cell proliferation and activation, and the secretion of immune-related cytokines.^85^ Polymorphisms of the OX40L corresponding gene (TNFSF4) have been especially correlated with SLE,^87–89^ and have a general correlation with SS,^90^ system sclerosis^91,92^ and sleep disorder narcolepsy.^93^ The inhibition of OX40 showed the potential for atopic dermatitis treatment in a phase 2a clinical trial.^94^ Each signaling pathway interacts with each other to form a complex and multidimensional signaling network to maintain immune homeostasis. Researchers tried to treat autoimmune diseases by targeting these pathways to inhibit the expansion of inflammatory effects.

## T cells and autoimmune disorders

T cells are the main cell type responsible for maintaining tolerance and play a key role in many autoimmune diseases. In this section, we describe autoimmune diseases that are characterized by inappropriate activation of autoreactive T cells and break of T cell tolerance. We review the clinical-related information and pathogenesis of T cell-mediated diseases including MS and T1D.

### Multiple sclerosis

#### Epidemiology, genetic factors, and environmental triggers

MS is an inflammatory demyelinating disease that affects the central nervous system (CNS) and is the most common cause of non-traumatic disability in young people.^95–98^ There are about 2.8 million people living with MS and a new patient appears every 5 min all over the world.^99^ The incidence rate for females is twice that of males, but the ratio can even reach 4:1 in some countries.^97,99^ MS is a complex autoimmune disease with substantial heterogeneity among patients. Researchers have discovered that the HLA-DR15 haplotype may be the major consideration for MS risk genetically.^100^ Besides, another large genetic research established a reference map about susceptibility genes of MS based on big data processing, which includes 200 autosomal susceptibility variants outside the MHC region, 32 variants in the MHC region and 1 variant in chromosome X.^101^ The MS risk-related gene map can help us to continue to deeply investigate the mechanisms of MS. For environmental factors, researchers have shown that the commensal microbiota in the human intestine may affect the occurrence of MS, based on their role in maintaining immune cell homeostasis. Disturbance in the composition of microbiota may trigger MS.^102,103^ Some researchers also point that EBV infection is essential for MS, and there is evidence to support that the MS prevalence of people with EBV infection is 32 times more likely than that of other virus infections.^104^ The mechanism of EBV infection to cause MS is still not clear, current studies suggest the role of molecular mimicry mentioned above in causing the break of immune tolerance and the development of autoimmune disorders.^105^ People in higher latitudes are more likely to have MS and researchers inferred that stronger ultraviolet light in high latitudes will affect the level of Vitamin D which can further affect the onset and prevalence of MS.^106,107^ Obesity and smoking have also been reported to have a certain correlation with MS.^108,109^

#### Clinical manifestation and diagnosis

The majority of MS patients will experience a relapse remission phase called Relapsing-Remitting MS (Rel-Rem MS, RRMS) characterized by acute relapses followed by partial recovery. Over time, about 80% of patients with RRMS will develop to the secondary process called secondary-progressive MS (SPMS) at which time the patient’s condition will deteriorate suddenly.^97^ Primary-progressive MS (PPMS) accounts for around 15% of MS patients which is characterized by a progressive disease course without a relapsing-remitting phase onset. The clinical manifestations of MS include cognitive impairment, motor impairment, fatigue, visual disorders and sensory disorders.^110,111^

For MS diagnosis, the combination of clinical, imaging and laboratory evidence is used. The diagnosis of MS via the detection of CNS lesions by T2-weighted scans or the contrast agent gadolinium from magnetic resonance imaging (MRI) and some other diagnostic methods are in continuous development such as positron emission tomography (PET) imaging technology.^112–114^ In addition, the detection analysis of cells and IgG antibodies, protein concentration, pleocytosis, and some immune cells in cerebrospinal fluid (CSF) and CSF oligoclonal bands are equally important to provide evidence for clinical diagnosis of MS.^115,116^ However, there are still no clear blood biochemical indicators available that can reflect the development of MS accurately. In addition, temporal and spatial development of clinical manifestations can provide the diagnostic basis for MS.^116^

#### Immune dysregulation in MS

**Fig. 3:**
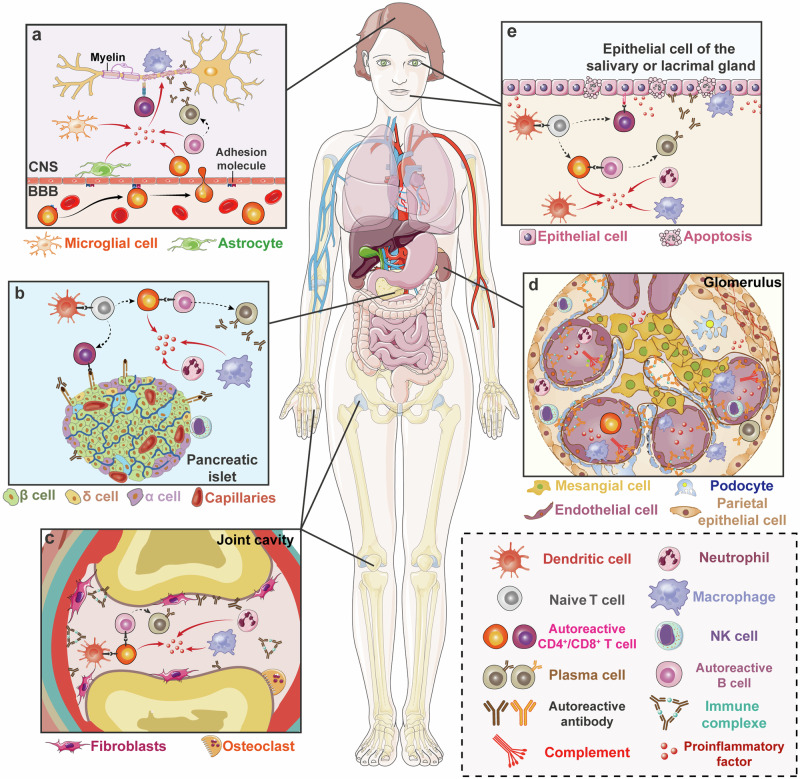
Pattern diagram of some typical autoimmune diseases. **a** Mechanism diagram of MS. Autoreactive T cells enter the CNS through the adhesion molecules on the BBB and trigger local inflammation of the CNS which causes the demyelination reaction and neuronal cell death. **b** Mechanism diagram of T1D. DCs induce the generation of autoreactive T cells which promote the local inflammation of the pancreas and cause the death of pancreatic β cells which lead to impaired glucose metabolism. **c** Mechanism diagram of RA. After the activation of induced autoreactive T cells by DCs, various immune cells in the joint cavity begin to execute abnormal programs and fibroblasts will proliferate. The autoreactive antibodies released by B cells can form immune complexes which further expand local inflammation. It ultimately causes the death of osteocytes and osteoarticular injuries. **d** Mechanism diagram of SLE. It most often involves the kidney, and the pathological change is similar to RA. Immune complexes and complement will deposit in the glomerulus and promote the inflammatory reaction which causes kidney damage finally. **e** Mechanism diagram of SS. The mechanism of abnormal activation of immune cells is similar to the aforementioned diseases. But it mainly occurs in salivary and lacrimal glands which leads to the epithelial cell death and loss of the function. (Part of the figure was modified from Servier Medical Art(http://smart.servier.com/), licensed under a Creative Common Attribution 4.0 Generic License. (https://creativecommons.org/licenses/by/4.0/)

The cause of MS remains elusive. The development of MS may start from the dysregulation of peripheral immune tolerance or CNS intrinsic events. The autoreactive T cells activated at peripheral traffic to the CNS through the blood-brain barrier (BBB) via some adhesion molecules (VCAM-1 and ICAM-1) to attack the myelin sheath formed by oligodendrocytes in CNS, meanwhile trigger more immune-activated cells infiltration to CNS, up-regulate the inflammatory signaling pathways and induce more inflammatory cytokines. Myelin-reactive T cells can migrate into the bone marrow in a CXCR4-dependent manner to skew hematopoietic stem cells (HSCs) toward myeloid lineage and augment CNS inflammatory injury and demyelination.^117^ Researchers suggested that the activation of memory B cells can drive the autoproliferation of Th1 brain-homing cells via HLA-DR.^118^ This work provides an explanation for the efficacy of anti-CD20 therapy for MS. Epitope spreading causes the change of autoantigens during the disease progression and gives rise to pathogenic T cell clones that evade regulation by Treg cells and trigger more damage.^95,97^ This is also the key and difficult point of treatment (Fig. 3a).

The cells involved in MS include T cells, B cells, APCs, myeloid cells and some glial cells. Th1 and Th17 play main roles in attacking the myelin sheath specifically by secreting inflammatory-related cytokines and CD8^+^ T cells contribute to disease pathogenesis via a FasL-dependent mechanism that promotes lesion formation.^119–121^ B cells secrete antibodies or inflammatory cytokines to attack the myelin sheath. Besides, other inflammatory cells also secrete proinflammatory factors such as IFN-γ, TNF-α, IL-17, IL-23, etc. Foxp3^+^CD4^+^ T cells, IL-10^+^ T cells (TR1),^122,123^ and some regulatory B cells (Bregs) can secrete IL-10, TGF-β, IL-35, and other anti-inflammatory factors.^124,125^ Astrocytes are the initiators to create the inflammatory environment by generating MMPs, ROS, TNF-α, and RNS. In this pathological environment, CNS will be severely damaged and eventually lead to disease-related features^97,126–128^ (Fig. 3a).

### Type I diabetes

#### Epidemiology, genetic factors, and environmental triggers

Type I diabetes (T1D) is a common autoimmune disease closely related with pathological T cell activation which is characterized by T cell infiltration into pancreatic islets and triggers immune responses against β-cell antigen.^129,130^ Approximately 8.4 million patients suffer from this disease worldwide, and the total incidence rate is increasing by 2–3% annually.^131,132^ According to the Markov model approach, researchers predicted that the affected populations will reach about 13.5–17.4 million in 2040.^131^ Availability and affordability of medicines for diabetes are poor in lower-middle-income countries.^133^ Although T1D can be diagnosed in any age group, the common population are children and adolescents. The peak manifestation period of T1D is between the ages of 5 and 7, as well as the pre-puberty period.^134,135^ Unlike typical autoimmune diseases, T1D is not biased towards females in terms of gender and the incidence rate of males will be slightly higher.^136^

T1D is a typical polygenic hereditary disease and susceptibility of T1D is strongly associated with genes that encode classical HLA. HLA DRB1*0301-DQA1*0501-DQ*B10201 (DR3) and HLA DRB1*0401-DQA1*0301-DQB1*0301 (DR4-DQ8) have been shown to increase disease susceptibility by 50%. In addition, DRB1*1501-DQA1*0102-DQB1-0602 (DR15-DQ6) appears to be protective.^137–139^ MHC-I-related genes also have an impact on the development of diseases and the mechanism of the effect is independent of MHC-II. More than 60 genes outside the HLA loci region such as CTLA4, PTPN22, KIR, VNTR, IL2RA, INS, etc. also contribute to T1D.^137,139^ Environmental triggers, daily dietary habits, and related enterovirus infection are associated with the development of T1D.^140^ Susceptibility factors such as obesity, vitamin D levels, virus infection, and human microbiota are similar to other autoimmune diseases.

#### Clinical manifestation and diagnosis

Fatigue, weakness, and lethargy will run through the entire disease process for T1D patients. If not treated in a timely manner, it will trigger a series of microvascular complications such as blindness, kidney failure, amputation, terminal sensory impairment, myocardial infarction and cerebral infarction.^141^

For patients with classical symptoms, diagnosis is based on the fasting blood glucose above 7 mmol/L, and 2-h plasma glucose value (2-h PG) above 11.1 mmol/L during the oral glucose tolerance test. Besides, it may be diagnosed by A1C concentration above 48 mmol/mol.^142^ Acute onset of T1D should be diagnosed by plasma glucose rather than A1C assay. C-peptide concentration as the marker of endogenous insulin level can serve as a diagnostic reference. However, it’s not enough to distinguish type I and type II diabetes only via the above detection methods.^129^ Biomarkers such as insulin autoantibodies and glutamic acid decarboxylase autoantibodies should be detected.^143,144^ T1D is defined by the presence of one or more such biomarkers.

#### Immune dysregulation in T1D

The analysis of biomarkers indicating the process from disease susceptibility to active immunity, and finally to the loss of autoimmune regulation, leads to the comprehensive understanding of T1D disease pathogenesis.^143^ The onset of T1D is considered to be the presentation of β-cells-related peptides via APCs to naïve T cells in pancreatic lymph nodes. These naïve T cells contacted with APCs escaped to the periphery because of the abnormal genetic variation mentioned above when they undergo both positive and negative selection in thymus.^145^ The activated T cells will further differentiate into functional effector and memory T cells. Part of CD4^+^ T cells will assist B cell differentiation to plasma cells to produce multiple anti-β-cells antibodies and others will continue to secrete inflammatory cytokines.^145^ Various myeloid inflammatory immune cells can enter pancreatic islets via gradient changes of chemotactic factors and attraction of numerous inflammatory cytokines for inflammatory environment expansion.^146^ More CD8^+^ T cells can release killing factors such as perforin and granular enzymes when they contact with β-cells directly.^147^ These immune cells will lose the immune regulation function gradually with the T1D development.^129,130,148^ The entire pancreatic islet shows immune infiltration and overall pancreatic manifestations are reduced volume, morphological atrophy and loss of secretion function^149^ (Fig. 3b).

## Autoantibodies and autoimmune disorders

Autoimmune diseases mainly mediated by antibodies tend to be more like systemic syndrome caused by immune system disorders. From the initial immune imbalance of a single organ or tissue, almost all kinds of organs in the body will be affected because of the occurrence of epitope diffusion. Here we review the clinical-related information and pathogenesis of autoantibodies-related diseases including RA, SLE and SS.

### Rheumatoid arthritis

#### Epidemiology, genetic factors, and environmental triggers

RA is a common systemic autoimmune disease and chronic inflammatory arthritis characterized by symmetric and polyarticular pain. RA mainly accumulates synovium and surrounding soft tissue at the synovium.^150–152^ The incidence of RA varies widely across the world which is reflected in the higher incidence in Europe and North America, and lower incidence in Southeast Asia region.^153^ The age-standardized prevalence and incidence increased by 7.4% and 8.2%, respectively.^154^ All ages are at RA risk, but the risk increases significantly after the age of 40.^155^ The male-to-female sex ratio also increases with age from 1:2 in the young to 1:4 in the old, which may be caused by the decline of estrogen levels after menopause in women.^153,156^

HLA-DR locus is the most important genetic risk factor for RA. Researchers found the key 5 amino acid sequences (70–74) of the HLA-DRβ chain called a shared epitope.^157,158^ Other genetics regions such as PTPN22, PADI4 and TNFRSF11A in non-MHC regions also contribute to the RA occurrence even if the contribution is not particularly significant.^159–161^ Besides, epigenetic modifications such as DNA methylation will increase RA susceptibility.^162^ For environmental factors, smoke seems to be the most important for RA.^163^ The reason may be that exposure to cigarette smoke promotes the pulmonary mucosal and draining lymph nodes prior to inflammation and then induces immune disorder inside the organism.^164,165^ Besides, some infection factors such as EBVs, retroviruses and bacteria especially in the oral cavity and the interaction of many microorganisms influence RA occurrence, but the specific mechanism is still unclear.^166–168^ Obesity and sodas are also important for RA.^169^ However, it is worth noting that alcohol intake seems to provide protection against RA, and some groups considered this may be related to the change of the microbial structure composition by alcohol.^170^ Some trials also demonstrated that long-term supplements of Vitamin D and omega-3 fatty acids can decline the RA incidence.^171,172^

#### Clinical manifestation and diagnosis

For most patients, the clinical symptoms show the gradual pain and swelling of joints early and the chronic inflammation of almost whole-body joints at later RA stages. The wrists and finger facet joint usually have an obvious manifestation at an early stage. With the development of RA, the large joints such as shoulders and knees will show corresponding symptoms. Affected joints will become bloated, and even develop into deformity and cause limited movement in severe cases.^150^ The affected joints will progress from active inflammation to irreversible lifelong damage without treatment. Morning stiffness is the characteristic performance of RA, and it usually lasts 30 min or longer time with fatigue and weakness simultaneously. Serious patients may have a high level of C-reactive proteins (CRP) and erythrocyte sedimentation rate (ESR), and some patients may have a fever and weight loss. Furthermore, RA can increase the incidence of cardiovascular disease, and it is mainly manifested in a functional lesion of the coronary artery. Some patients also developed pulmonary fibrosis and inflammation of the respiratory system with RA expansion.^173–175^

The American College of Rheumatology (ACR) and the European Alliance of Associations for Rheumatology (EULAR) revised the diagnostic criteria in 2010. The new diagnosis was made by the overall score of the 4 dimensions which include the number counting of joint involvement, rheumatoid factor (RF) antibody and anti-cyclic citrullinated peptide antibodies (ACPAs) titers in serum, CRP and ESR of acute phase reactants and whether the duration of symptoms lasts for 6 weeks. When the score is more than 6, RA can be confirmed clinically.^176,177^

#### Immune dysregulation in RA

A variety of autoantibodies, mainly ACPAs and RF antibodies, are the initiators of this disease.^178,179^ The cell-cell interaction of specific immune cells within the synovium is the basis for RA occurrence. APCs represented by DCs present the RA-related antigens such as citrullinated peptides to T cells with the major phenotypes as CD4^+^ PD1^+^CXCR5^-^, and they are also called peripheral helper T (Tph) cells that generate IL-21 primarily within the synovium.^180^ Besides, some CD8^+^GZMK^+^ T cells also appear to generate IFN-γ.^181^ Tph cells can assist B cells to differentiate into plasm cells and generate a large number of antibodies along with IL-6 and GM-CSF to attack the tissue in the synovium. In this process, macrophages, neutrophils and other myeloid cells can provide the inflammatory environment. Numerous fibroblasts also emerge under the action of TNF-α, IL-12, IL-13, IL-17 and TGF-β, and amplify inflammatory effects.^182–184^ Monocytes will further differentiate into osteoclasts to release related proteases for bone erosion and cartilage loss. Researchers found a distinct population of CX3CR1^+^ tissue-resident macrophages that exert immune regulatory function by maintaining a tight-junction-mediated barrier and restricting inflammation.^185^ These multiple pathways and mechanisms expand into a systemic autoimmune response without effective treatment^151,186,187^ (Fig. 3c).

### Systemic lupus erythematosus

#### Epidemiology, genetic factors, and environmental triggers

SLE is an autoimmune disease characterized by producing anti-nuclear autoantibodies and causing the immune complex deposition in various organs, and it leads to chronic and systemic diffuse connective tissue disease that mainly affects young women.^188–190^ The overall global prevalence and the incidence of SLE are about 0.3–0.5% and 0.0022–0.0231%, respectively.^188^ The annual age-standardized mortality rate of patients is higher than many other autoimmune diseases, and about 2.7 deaths per million inhabitants in 2014.^191^ The mortality rate of women is much higher than that of men. Black, Asian, and Spanish populations have a higher risk than the white population for SLE and the clinical manifestation of diseases is more serious.^191–194^ It is worth noting that about 90% of patients are women, and most of them are of childbearing age and presenting diversity in SLE performance can significantly affect fertility function.^195^

HLA-II gene region is the susceptible locus of SLE, and HLA-DRB1 has the strongest correlation with SLE. Studies have shown that HLA-DRB1*03:01 is related with the generation of anti-Ro and anti-La autoantibodies and HLA-DR3 has a strong connection with anti-dsDNA antibodies. A high-density case-control single nucleotide polymorphism research in the MHC region identified the independent and interacting sites of HLA-DPB1, HLA-G and MSH5.^196^ Besides, mutations in complement pathway-related genes are a high risk for SLE because of the obstacle to cleaning the cellular debris. Monogenic influence on SLE to cause high-IFN levels is also undeniable, and these monogenic groups include DNASE1/DNASE1L3, PRKCD, TREX1, STING, SAMHD1, etc.^196^ Epigenetic modification is also an important genetic reason.^197^ Like many other autoimmune diseases, smoking and EBV infection can induce the pathogenesis and moderate drinking provides a protective mechanism.^198,199^ The difference is that mercury and silica exposure are important environmental factors for SLE because of their function as an adjuvant to induce the transcription of proinflammatory cytokines and T-cell responses.^200,201^

#### Clinical manifestation and diagnosis

The clinical features of SLE are heterogeneous and various organs are affected. The patients usually have constitutional symptoms and fevers, and many patients also exhibit skin and mucosal symptoms such as butterfly erythema, mucosal ulcer (usually appearance in oral and nasal cavities) and alopecia. Butterfly erythema is a typical symptom appearing as red patches located on the bridge of the nose or both sides of the cheekbones.^190,192^ Many patients have joint and bone pain complications similar to RA, which are also symmetrical and have morning stiffness. Some patients also have chest, pericardium, and peritoneal fluid once the serosal inflammation progresses to a certain extent. Lupus nephritis is a major visceral manifestation of RA, the patients will experience hematuria, proteinuria, and possible systemic edema at last.^202^ Besides, SLE can affect the cardiovascular system to cause pericarditis, endocarditis and coronary artery lesions, the respiratory system to cause pulmonary arterial hypertension and pulmonary fibrosis, and the digestive system to cause pancreatitis and a series of intestinal diseases.^190,203,204^ Therefore, early identification and intervention are necessary to prevent serious and irreversible pathological damage.

EULAR and ACR developed new classification criteria in 2019 that include positive antinuclear antibodies (ANA) followed by 7 clinical (constitutional, hematological, neuropsychiatric, mucocutaneous, serosal, musculoskeletal, renal) and 3 immunological (anti-phospholipid antibodies, complement proteins and SLE-specific antibodies inspection) items.^205^ Anti-nuclear antibodies at a titer of ≥1:80* on HEp-2 cells or an equivalent positive ANA test should be used as the entry criterion.^205^

#### Immune dysregulation in SLE

The pathogenesis of SLE is complex, with non-immune cells, innate immune responses and adaptive immune responses participating in the disease process. Endogenous nucleic acid combined with autoantibodies in the form of immune complexes (ICs) has the potential to drive the production of IFN-α in plasmacytoid dendritic cells which is pivotal in the pathogenesis of SLE.^206^ Besides, Janus kinase (JAK)-signal transducer activator of transcription (STAT) pathway and Bruton’s tyrosine kinase (BTK) pathway have been shown to play important roles in the pathogenesis of SLE.^207,208^ The inflammatory environment promotes adaptive immune response, and APCs dominated by DC can present autoantigens to T cells. These activated T cells further expand inflammatory response by releasing more inflammatory cytokines (TNF, B lymphocyte stimulator, etc.) and simultaneously assist in the activation of B cells.^209^ B cells undergo differentiation to plasma cells to produce massive autoantibodies and form complexes with numerous nucleic acids and related proteins. ICs can deposit and promote an intense inflammatory response to damage the corresponding organs and tissues, and ultimately lead to the development of SLE^206^ (Fig. 3d).

### Sjögren’s syndrome

#### Epidemiology, genetic factors, and environmental triggers

Sjögren’s syndrome (SS) is a systemic and chronic autoimmune disease characterized by inflammatory reaction of exocrine organs including but not limited to lacrimal and salivary glands that lead to the drying of the mouth, eyes, respiratory tract, and vagina eventually.^210–212^ The prevalence and incidence of SS is about 0.01–0.72% and 0.003–0.011% in the population, respectively.^210^ The gender difference and clinical features are obvious for SS, the ratio of female to male patients is about 10:1 and female patients have more serious clinical manifestations.^213^ A study for the epidemiology of SS in a French multiracial population discovered that the non-European race has a higher SS prevalence and disease profile than the European race, and another study discovered a higher prevalence for white females.^214,215^ Although SS can occur at all ages, children are rarely diagnosed and the population in 30–50 age are mainly diagnosed.^210^

HLA gene region is also the key to SS occurrence, and a recent review has summarized detailed research about the genetics and epigenetics of SS. Genes significantly associated with SS and exhibiting pathogenicity include HLA-DQA1, HLA-DQB1, HLA-DRA (rs115575857), HLA-DRB1 (rs116232857),^216^ HLA-B (rs2523607)^217^ and MICA (MICA*008)^218^ in MHC region, TNF (rs1800629),^219^ STAT4(rs10168266)^220^ and IL12A (rs485497)^216^ in non-MHC region. In addition, IKZF1 (rs4917129),^221^ OAS1 (rs10774671)^16^ and MAPT (rs7210219)^222^ may possess SS protective mechanisms. The epigenetic modification also affects the occurrence and development of SS.^223^ In addition to common environmental factors which can induce autoimmune diseases, silicone breast implants also lead to a high risk for SS.^224^ Virus infection seems to be particularly important for SS and many studies have demonstrated that EBV protein EBNA2 can bind with related high-risk sites of SS.^222^ Unlike other autoimmune diseases, smoking is not associated with the development of primary SS.^225,226^

#### Clinical manifestation and diagnosis

There is typical heterogeneity in the clinical manifestations of primary SS, similar to SLE, and the patients have various performances because of different organ involvement. Almost 85% of patients will have glandular symptoms manifested as ocular dryness (major symptom), ocular inflammation, oral drying (major symptom), dysphagia, pruritus in the ear canal, vaginal pruritus or dyspareunia. Approximately 50% of patients will have cutaneous features such as cutaneous vasculitis, including purpura and urticarial papules that depend on the condition of the blood vessel lesion.^215^ Some patients show the nonspecific phenomenon such as Raynaud’s phenomenon of skeletal muscle pain and fatigue.^227^ Almost half of primary SS patients can develop into systemic performance and invasion of the kidney, lung, liver, and other organs.^228^

SS can be diagnosed via a series of exocrine gland tests and laboratory examinations. Patients will have assessment tests such as unstimulated salivary flow rates, stimulated salivary flow rates, and salivary scintigraphy for evaluating the main salivary glands. Schirmer’s test I, Schirmer’s test II, and Corneal staining can be used to evaluate the lacrimal gland function.^210^ Autoantibodies detection is very sensitive and can be detected even 20 years before SS occurrence.^229^ Antinuclear antibodies (ANAs) are the most common for the majority of patients. Anti-RNA-related protein antibodies (anti-Ro/SSA antibodies) are representative of different clinical stages, histological changes and immunopathological changes. In addition, anti-La/SSB antibodies are also specific antibodies for SS patients.^230^ For laboratory abnormalities, the samples from SS patients show normocytic anemia, leukopenia, and thrombocytopaenia, and some advanced patients will show the elevation of visceral damage-related enzymes. Salivary gland biopsy is the most specific detection method, and clinical pathologists can make the final diagnosis of SS via checking the distribution and number of antibodies and lymphocyte infiltration.^231^

#### Immune dysregulation in SS

The pathogenesis of autoimmune epithelitis is an explanation for the immunopathology of SS. The TLRs molecular pathway activation of glandular epithelial cells such as salivary gland epithelial cells leads to the production of autoantigen that can be presented to immune cells. Furthermore, activation of TLR signaling leads to the upregulation of immune-competent molecules such as HLA molecules, FAS receptors and ligands, chemokines, and cytokines. Immune cells and inflammatory microenvironment create a circle of interaction between epithelial cells and immune cells that promotes the development of SS^210,223^ (Fig. 3e).

## New therapeutic strategies for autoimmune disorders

Here we mainly summarize antibody therapy, RNA interference (RNAi) therapy, and Hematopoietic stem cell transplantation (HSCT) therapy for autoimmune diseases. We review the outcome of these approaches and discuss their translational potential.

### Antibody therapy

#### Combination of targeted antibody therapies

It is undeniable that single antibody treatment may have some effect on autoimmune diseases, however, combined treatment may target two or more signaling pathways and achieve synergistic treatment effects.

In a 68-week phase II double-blind study for primary SS treatment (GSK study 201842, NCT02631538), researchers used the combination of belimumab and rituximab to achieve more effective results than single rituximab treatment. Almost all CD20^+^ B cells in salivary glands are exhausted and the phenomenon also occurs in peripheral CD19^+^ B cells simultaneously. The regulative effect is more intense and lasting for the combination of belimumab and rituximab than single rituximab treatment. In addition, there are no new side effects added.^232^ Another randomized controlled trial (ISRCTN: 47873003) tried belimumab after rituximab treatment mode for SLE patients via the score of the IgG anti-dsDNA antibody level in serum.^233^ Other groups also proved the low-dose rituximab and alemtuzumab combination treatment for autoimmune cytopenias can achieve a 100% overall remission rate and 58% complete response but there are still 6 patients developing infection (NCT00749112).^234^ Anti-CD22 monoclonal antibody conjugated with calicheamicin (anti-CD22/cal) and CTLA4-Ig combination therapy can suppress autoimmunity in NOD mice and prolong the allograft survival time.^235^ Recently, researchers in the Hospital for Special Surgery (New York) also conducted a clinical trial to detect the treatment effect of belimumab and rituximab combination in diffuse cutaneous systemic sclerosis (NCT03844061). However, not all combination therapies can have significant therapeutic effects, Atisha-Fregoso et al. demonstrated that the combination of belimumab and rituximab did not alleviate symptoms of general Lupus Nephritis patients (NCT02260934).^236^ The rituximab and alemtuzumab combination therapy trial (NCT03312907) for SLE by GlaxoSmithKline started in 2019.^237^ Regrettably, the result of combination therapy illustrates that it cannot improve disease conditions and even cause more serious infections.^238^

Compared with therapeutic measures of multiple antibody combinations, therapeutic monoclonal antibodies combined with some chemotherapeutics or other immunosuppressive biologics seem to be more widely applied. A study about Certolizumab pegol and methotrexate (MTX) combination treatment (NCT01519791) for RA showed a significant therapeutic effect without extra side effects compared with placebo + MTX.^239^ In some earlier studies, researchers also tried to treat relapsing MS with natalizumab plus IFNβ-1a (NCT00030966) and followed up on the patient’s recurrence and MRI images. Although the therapy results are encouraging, there are still unavoidable adverse reactions such as anxiety, congestion and edema.^240^ Glatiramer acetate and natalizumab combination also have significant therapeutic effects and are well tolerated.^241^ Ocrelizumab (200 mg) with MTX can reduce the development of RA, but ocrelizumab (500 mg) with MTX will lead to ascending levels of serious infections (NCT00485589).^242^ The type I interferon receptor antibody, anifrolumab, combined with oral glucocorticoids and mycophenolate mofetil (MMF) achieved some success in complete renal response (CRR), however, the incidence of herpes zoster in the combination group was twice that in the placebo group (NCT02547922).^243^ Belimumab with MMF or cyclophosphamide-azathioprine combination trial (NCT01639339) for Lupus Nephritis also confirmed the effectiveness of combination therapy.^244^ Rituximab and prednisone combination for warm autoimmune hemolytic anemia in adults (NCT01181154) showed more effective and safer than placebo with prednisone.^245^ Burmester et al. also specially studied the influence of the combined MTX dose on side effects and explained the correlation between dose effect and clinical efficacy (NCT01185301).^246^ Studies also propose less certain treatment effects in monoclonal antibodies combined with other immunosuppressive drugs. Rituximab + MMF + corticosteroids combination (NCT00282347) did not show more excellent therapy results compared with rituximab treatment alone.^247^

In sum, there is still uncertainty in antibody combination therapy, and no simple superposition of therapeutic effects through several targeted drugs and antibodies combination. Meanwhile, the drug side effects may be strengthened by medicine combination. In addition, a large number of clinical trials are needed to explore the dose of drugs used in combination therapy.

#### Bispecific antibodies therapies

**Fig. 4:**
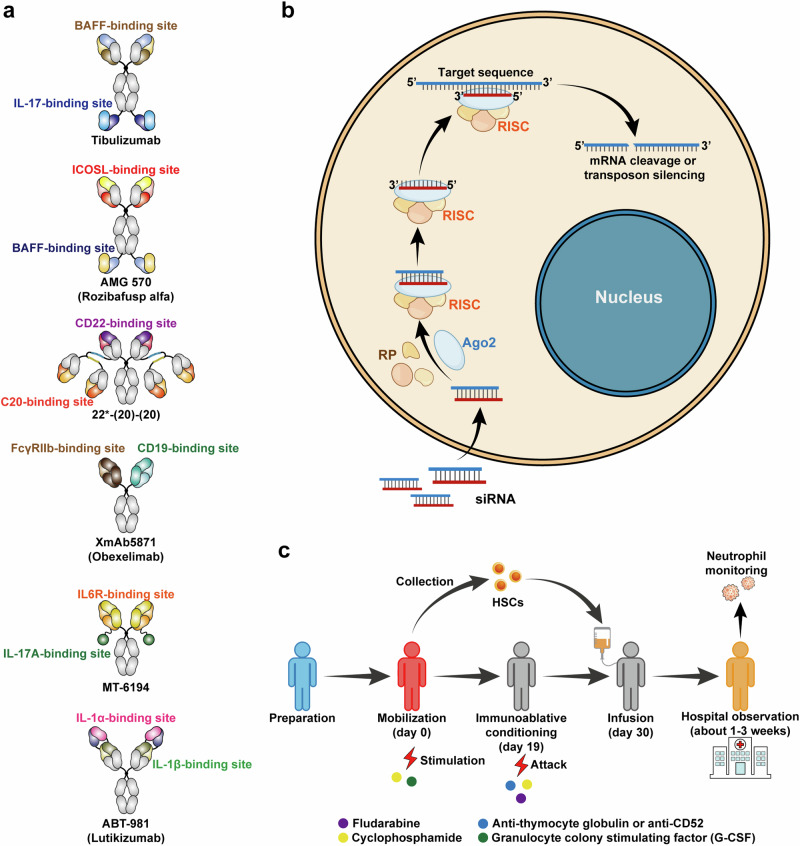
Other new therapeutic strategies to autoimmune diseases. **a** Some examples of bispecific antibodies in clinical trials. **b** The schematic diagram of intracellular mechanisms of siRNA. siRNA consists of a guide (antisense) strand and passenger (sense) strand. The former is a functional segment for siRNA and the latter is responsible for transportation and loading. siRNA can combine with RNA-induced silencing complex (RISC) consisting of Argonaute 2 (AGO2), trans-activation response RNA binding protein 2 and DICER1. After the degradation of the passenger strand, the target RNA sequence can be recognized by the guide strand. Eventually, it can induce the silence of the target RNA. **c** The schematic diagram of hematopoietic stem cell transplantation (HSCT). Before determining transplantation, transplanted patients should be identified. Besides, patients are monitored to prevent flares. Generally, G-CSF and cyclophosphamide (2–4 g/m^2^) plus uromitexan are applied to the mobilization of HSCs in patients. About 4 or 5 days after mobilization, we collect the peripheral blood stem cells by leukapheresis and these cells are CD34^+^ in general. The patients can be discharged and wait for the immune conditioning after 1 or 2 weeks. The conditioning process may last for about 10 days. Then HSCs can be infused back into the patients. Patients accepting HSCs are left to observe in the hospital until the neutrophil level returns to normal. After HSCs infusion, the patients’ lymphocytes may decrease extremely but their immune systems can rebuild

Bispecific antibodies (BsAbs) are a new class of antibodies that can identify two different antigens or two different epitopes of the same antigen (Fig. 4a). The successful generating of more than 100 BsAbs formats benefit from the significant advances in antibody engineering and antibody biology.^248^ Thanks to their strong multitargeting, high binding potency, bridging cell action, and cascade amplification effect,^249,250^ they have been applied to the treatment of complex tumors and autoimmune diseases.^251–256^

Bimekizumab which can selectively inhibit IL-17A and IL-17F simultaneously is the first BsAbs approved by the FDA in 2021. In two studies for the treatment of plaque psoriasis, adalimumab (NCT03412747) and secukinumab (NCT03536884) were compared with bimekizumab, respectively to evaluate the treatment effect of bimekizumab.^257,258^ bimekizumab showed non-inferior therapeutic ability to adalimumab in reducing symptoms and signs of plaque psoriasis but had adverse events including higher frequency of oral candidiasis and diarrhea.^257^ Besides, bimekizumab is also applied in moderate-to-severe plaque psoriasis (NCT03025542, NCT03410992),^259,260^ hidradenitis suppurativa (NCT03248531),^261^ RA (NCT02430909)^262^ and ankylosing spondylitis (NCT02963506, NCT03928704, NCT03928743),^263,264^ and has achieved good curative effects, but infections and infestations still persist.

Tibulizumab (LY3090106) is another novel tetravalent BsAb which can target and inhibit the B cell activating factor (BAFF) and IL-17, and it is synthesized by the link of anti-IL-17 single-chain variable fragment from ixekizumab and the anti-BAFF fragment from tabalumab^265^ (Fig. 4a). And in vivo mouse models and cynomolgus monkey, tibulizumab can effectively inhibit the development and survival of B cells for a long time in mouse models and cynomolgus monkey.^265^ Relevant clinical trials (NCT03736772, NCT01925157, NCT02614716) have been initiated, but no results have been disclosed yet.

Rozibafusp alfa (AMG 570) BsAb composed of the AMG 557 antigen providing anti-ICOSL sequence and the BAFF-binding peptides from AMG 623 linking with the C-terminus of AMG 557 heavy chain,^266^ can target and inhibit the BAFF and ICOSL (Fig. 4a). The treatment effect is more significant than single inhibitor in mouse NZB/NZW lupus model and arthritis (CIA) model. It can also inhibit the development of B cells in cynomolgus monkeys.^266^ Clinical studies (NCT02618967, NCT03156023) have been initiated to investigate the pharmacokinetics (PK) and pharmacodynamics (PD) of rozibafusp alfa.^267^

Anti-CD22/CD20 bispecific hexavalent antibody (bsHexAb), 22*-(20)-(20) is developed by Rossi et al., which is composed of Ck-AD2-IgG-epratuzumab (anti-CD22) and two dimeric CH1- DDD2-Fab-veltuzumab units (anti-CD20)^268,269^ (Fig. 4a). This BsAb may be inspired by the previous BsAb therapy for lymphoma.^270,271^ The researchers have tried to treat SLE with 22*-(20)-(20), and demonstrated the enhanced trogocytosis resulting in reductions of many B cell surface marker levels. In addition, the 22*-(20)-(20) used alone showed a better treatment effect than the combination therapy of the two parental antibodies.^269^

Notably, many BsAbs such as obexelimab (XmAb5871) targeting CD19 and FcyRIIb to inhibit B cells line,^272–274^ MT-6194 targeting both IL-17A and IL-6R to inhibit the development of inflammatory environment^275^ (Fig. 4a), JNJ-61178104 targeting TNF and IL-17A^276^ and romilkimab (SAR156597) targeting both IL-4 and IL-13^277^ have been generated and tested in clinical trials. Clinical trials about these BsAbs reflect the broad clinical application potential (NCT02758392, NCT02725515, NCT02725476, NCT02921971).^278–281^

Compared with application in tumor therapy, the research of BsAbs in autoimmune diseases is still in its infancy, and there are many challenges. In a clinical trial of lutikizumab (ABT-981)^282^ (Fig. 4a) for the treatment of arthritis with synovitis (NCT02087904), the pain and arthritis symptom improvement are not obvious.^283,284^ Another phase II clinical trial about SAR156597 for idiopathic pulmonary fibrosis (NCT02345070) has also failed.^285^ Although the results of clinical trials might vary, BsAbs still have many advantages and offer new therapeutic options for autoimmune diseases.^286^

### RNA interference therapy

RNAi was first discovered in Caenorhabditis Elegans by Fire and Mello in 1998.^287^ After that, researchers further studied these small mRNA (sRNA) and found small-interfering RNAs (siRNAs).^288^ Although there are many sRNA types including siRNAs, microRNA (miRNA) mimics, short hairpin RNAs (shRNAs) and Dicer substrate RNAs (DsiRNAs), the research on siRNA is more in-depth and shows more direct effects in translation.^289–291^ Hence, in this review, we emphasize the siRNA application for autoimmune diseases. siRNA usually is 15–30 bp in overall length. siRNAs can trigger efficient target gene silence by inhibiting mRNA translation and promoting mRNA degradation (Fig. 4b). Pharmaceutical companies have been devoted to developing the siRNA therapeutics and major breakthroughs were being made that paved the way to successful clinical translation.^292,293^ In 2018, the FDA approved the first liposome complex for siRNA binding (Patisiran) for the treatment of a rare disease called hereditary transthyretin-mediated amyloidosis (hATTR).^294–296^ Indeed, the rapid development of siRNA is benefit from lipid nanoparticles (LNPs) technology progress and related nucleic acid modification methods.^297–299^ Researchers also use siRNAs for the treatment of autoimmune diseases and achieved some progress.^300,301^

Herman et al. delivered siRNA based on the LNP system to two types of mouse models of RA for hnRNP A2/B1 silence and downregulate the expression of proinflammatory cytokines in macrophages.^302^ The noncovalent binding of siRNA targeting the p65 subunit of NF-κB (p5RHH-p65) and melittin-derived cationic amphipathic peptide can also control inflammation and protect the integrity of cartilages in RA.^303^ Other groups also tried PEG-PLL-PLLeu nanoparticle,^304^ polycaprolactone-polyethylenimine (PCL-PEI)/polycaprolactone-polyethyleneglycol (PCL-PEG),^305^ folate conjugated liposome-based hybrid carrier,^306^ etc., to deliver siRNA targeting NF-κB for the treatment of autoimmune disorders.

Lee et al. designed a nanocomposite composed of poly-siRNA targeting TNF-α and thiolated glycol chitosan (tGC) for RA treatment. The related inflammatory genes were effectively silenced in the macrophage stimulation culture test and mouse RA model.^307^ Besides, Different nanomaterial carriers are used to deliver the siRNA targeting TNF-α including Lipid-polymer hybrid nanoparticles (LPNs),^308^ degradable cationic polymer (PDAPEI),^309^ sheddable PEGylated solid-lipid nanoparticle,^310^ folate-PEG-chitosan DEAE nanoparticle,^311^ etc.

Poly-siRNA targeting Notch1 combined with tGC also has good performance in RA.^312^ In addition, siRNA is designed to target complement fragment 5 (C5),^313^ MMP-9,^314^ BTK,^315^ IFN regulatory factor 5 (IRF5)/ B cell-activating factor (BLYSS)^316^ and other inflammation-related genes.

Currently, research about siRNA for the treatment of autoimmune diseases has been mainly focused on RA, it is essential to investigate its potential treatment effects on other autoimmune diseases. With the rapid development of targeted drug delivery technology, siRNA-based therapy will undoubtedly be used to treat many other diseases.

### Hematopoietic stem cell transplantation

As previously discussed, the fundamental mechanism of autoimmune diseases is the break of autoimmune tolerance because of the environment and genetic factors. HSCT provides a treatment option to restore immune tolerance by replacing or resetting immune responses.^317^ During the immune reconstitution process, NK cells and B cells recovering faster than T cells, with CD4^+^ T cells recovered slowly compared to CD8^+^ T cells based on a study in MS patients after HSCT transplantation.^318^ The pre-existing T cells with pathological and autoimmune reactions will be replaced by newly formed T cells.^319^ After autologous HSCT transplantation in MS patients, B cells shifted from a predominantly transitional to naïve phenotype, and memory B cells recovered slowly with reduced repertoire diversity.^320^ Altogether, these processes can quench the pre-existing autoimmune responses and reestablish immune tolerance. However, complete deletion of all autoimmune pathogenic cells is impossible, and immune cells with regulatory capacity control the homeostasis of the repopulated immune system.^318^ Tregs play an important role in balancing the body’s immune axis.^321–323^ Besides, other cells represented by tolerogenic DCs (tolDCs) with tolerance characteristics have beneficial roles.^324,325^ TolDCs have enormous potential for the treatment of autoimmune diseases due to their ability to induce immune tolerance.^326–328^ These tolDCs express low co-stimulatory molecules and high levels of immunosuppressive membrane surface molecules including programmed cell death ligand (PD-L1)^329^ and inhibitory Ig-like transcripts (ILTs),^330^ which leads to the T cell clonal anergy and expansion of regulatory T cells eventually.^327^ In the antigen-specific treatment of autoimmune diseases, researchers regard induced tolDCs as a standard of treatment success and we will discuss them in more detail later.

The study of bone marrow transplantation for improving RA in rat models seems to be groundbreaking to HSCT therapy.^331^ Afterwards, related technologies developed rapidly and researchers have applied HSCT to various autoimmune diseases. From the European Society for Blood and Marrow Transplantation (EBMT) autoimmune diseases working party database, we can acquire the earliest therapy information started in 1994.^332^ In 1996, Tamm et al. reported the first treatment with HSCT for autoimmune disease.^333^ In 1997, Fassa et al. reported the first results of the treatment with HSCT for MS and preliminarily verified its feasibility.^334^

Autologous HSC may be derived from peripheral blood or bone marrow and the process is as follows^335,336^ (1) Mobilization of stem cells by treatment with cyclophosphamide and granulocyte colony-stimulating factor (G-CSF). Stem cells can be collected 4–5 days after the treatment. (2) Conditioning. 1 or 2 weeks after cell collection, the patients will accept the immunoablative conditioning including anti-thymocyte globulin and cyclophosphamide. The different ways will be implemented in different individual patients. (3) Infusion of autologous CD34^+^ stem cells and hospitalized for observation. Patients will continue to be hospitalized to prevent the sudden occurrence of adverse events after infusion for 1–3 weeks until the recovery of neutrophil numbers (Fig. 4c).

We mainly describe the therapeutic effect and development of HSCT in MS. In 2006, a retrospective survey of 183 MS patients from EBMT reported 5.3% transplant-related mortality (TRM) and 63% of MS patients improved the disease development or stabilized the mental state during the median follow-up of 41.7 months.^337^ Afterwards, a group reported that the HSCT can be effective for aggressive MS failing to respond to conventional treatment according to the Italian multi-center experience.^338^ In 2015, Burt et al. reported a significant improvement in the quality of MS patient life scores and the significant reduction of MRI T2 lesion area.^339^ Compared with standard immunotherapy, HSCT therapy promotes the continuous improvement of active secondary progression.^340^ Compared with alemtuzumab for RRMS, HSCT also seems to have more treatment feedback but it also leads to more adverse events in the first 100 days after transplantation in an observational study (NCT03477500).^341^ Similar results also appear in the comparison of HSCT with Fingolimod and natalizumab.^342^ A long-term clinical outcome and an observational cohort study in Sweden also affirmed the role of HSCT for most MS patients with certain efficacy and safety.^343,344^ However, a recent matched observational study did not support the use of autologous HSCT to control disability in progressive MS with advanced disability and low relapse activity.^345^

HSCT might also become a treatment option for other autoimmune diseases. It has been reported that children with refractory juvenile idiopathic arthritis (JIA) gradually recovered after reduced toxicity conditioning HSCT therapy. In this report, all the patients alleviated disease progression and improved their quality of life, 11 children of them even achieved complete drug-free remission.^346^ A clinical trial (NCT00742300) reported the disappearance of pathogenic dsDNA and resetting of the adaptive immune system, the regeneration of Foxp3^+^ Tregs from thymus in refractory SLE patients accepting HSCT after depletion of pre-immune system.^347^ Recently, researchers also found that HSCT favorably changed the antibody reservoir in systemic sclerosis patients.^348^ The C-peptide levels also increased significantly and most of the patients achieved insulin independence under good control of blood sugar level after HSCT,^349^ but a report demonstrated 52% of patients experienced adverse effects despite a complete immune system recovery.^350^ This study suggested an urgent need for safer HSCT options.

It is undeniable that there are many adverse effects of HSCT in autoimmune disease treatment. Researchers must consider the possible infertility, early menopause and heart damage in MS and systemic sclerosis patients.^335,351,352^ The use of immunosuppressants will also increase the risk of various infections and malignancies.^335,353^ Incomplete rearrangement of immune cells after HSCT will lay a hidden danger for the recurrence of autoimmune disease. A report showed nearly 10% of secondary autoimmune disease after HSCT.^354^ Besides, improved risk estimates and supportive care are particularly important for patients who received allogeneic HSCT.^355–357^ Nevertheless, the HSCT is still a reasonable option for the treatment of autoimmune diseases based on its capability to reset or rebalance the immune system to restore immune tolerance.

## Emerging therapeutic strategies based on antigen-specific immunotherapy

**Fig. 5:**
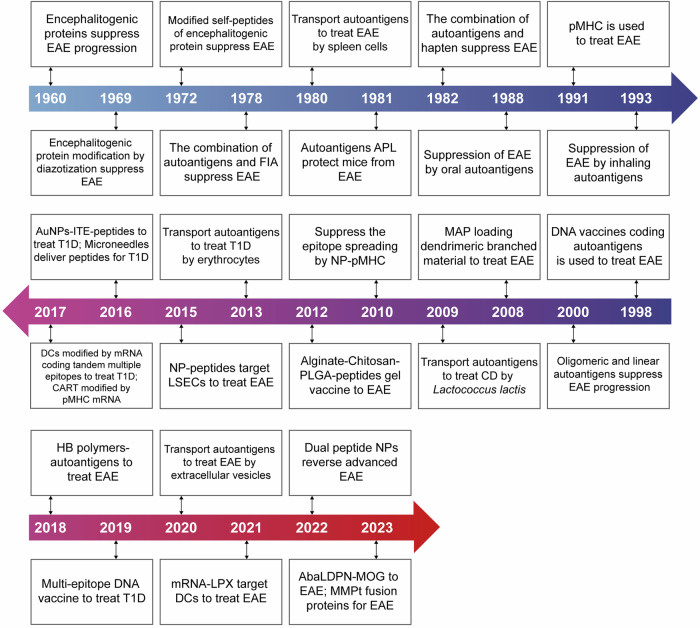
Timeline of the significant advances in the field of antigen-specific therapy for autoimmune diseases. In 1960, researchers discovered that encephalitogenic protein can suppress EAE progression. Then researchers tried to use modified autoantigens or MHC conjugated autoantigens to treat animal models of autoimmune diseases. In 1998, researchers have tried to use the DNA coding autoantigens to treat EAE. Afterward, the application of nanomaterials gradually emerged in autoantigens transportation, and antigen-specific therapy has experienced a rapid development over the past 20 years. Some researchers also tried to apply the combination of immunosuppressive factors, autoantigens, and nanoparticles for treatment. In 2021, mRNA-LNP technology has been applied for the first time in autoimmune disease models

Due to the fact that antigen-specific immunotherapy can target the disease-causing immune cells without suppressing the whole immune system, there has been an urgent need to develop new immunotherapies that induce long-term antigen-specific immune tolerance for the treatment of autoimmune diseases.^358^ Although significant advances have been made in this field, successful clinical application is still limited. Here we will discuss the current strategies developed in this field, and highlight the recent advances in the use of nanomaterials and mRNA vaccine techniques to induce antigen-specific immune tolerance. Besides, we also provide a timeline to summarize the significant advances in the field of antigen-specific immunotherapy for the treatment of autoimmune diseases based on MS and T1D (Fig. 5).

### Autoantigen-based therapies

#### Whole antigen or modified peptides

**Fig. 6:**
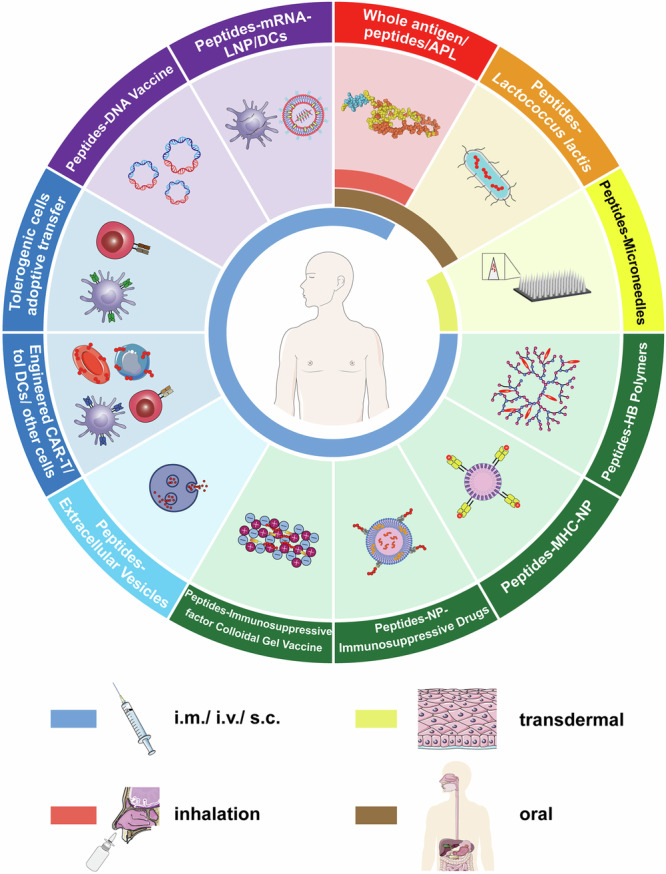
Approaches to deliver autoantigen for the treatment of autoimmune diseases. (1) Whole antigens, peptides, and APL are administered through subcutaneous injection, intravenous injection, intramuscular injection, oral and inhalation. (2) Autoantigens are transported by microbes such as *Lactococcus lactis*. (3) Microneedles loading antigens target DC cells in the skin. (4) Autoantigens are delivered by hyperbranched polymers. (5) Nanoparticles for delivering autoantigen or pMHC; (6) Combination of autoantigen, Nanoparticles, and immunosuppressive drugs. (7) Gel vaccine with immunosuppressive drugs. (8) Autoantigen transported by extracellular vesicles. (9) Engineered cells modified by autoantigen specificity. (10) Autoantigen-specific tolerogenic cells adoptive transfer. (11) Gene therapies based on DNA-plasmid coding autoantigens. (12) Gene therapies based on mRNA coding autoantigens. Abbreviations: i.m.= intramuscular injection; i.v. intravenous injection, s.c. subcutaneous injection. (Part of the figure was modified from Servier Medical Art(http://smart.servier.com/), licensed under a Creative Common Attribution 4.0 Generic License. (https://creativecommons.org/licenses/by/4.0/)

It has long been known that the damage of CNS can be prevented in animals by the administration of a mixture of encephalitogenic substances before the experimental autoimmune encephalomyelitis (EAE) model establishment. In 1960, SHAW et al. found that the combination of Freund’s adjuvants and encephalitogenic proteins extracted from the homologous brain can suppress the EAE progression, meanwhile, the suppressing effect is closely related to protein injection dose.^359^ They attributed this phenomenon to the specific desensitization, deflection, antibody neutralization reaction, or disability of antibody-forming mechanisms^359^ (Fig. 6). Early studies have shown that the combination of Freund incomplete adjuvant and myelin basic protein (MBP) inhibit EAE.^360^ However, the administration of MBP whole antigen has been shown to be ineffective treatments or major exacerbations have emerged both in clinical trials and in animal models.^361,362^ For T1D, insulin administration has been shown to prevent NOD mice from developing the disease.^363^ Multiple clinical trials using insulin immunotherapy have been conducted to prevent or treat T1D, but the results are still uncertain.^364–367^

The mechanism of antigen-specific immunotherapy is through induction of immune tolerance by injection of autoantigens with high and repeat dose that leads to T cell anergy or results in RICD and generation of Tregs.^16,368^ The RICD process is closely related to TCR recognizing antigens and FAS-FASL inducing apoptosis and it is an antigen-specific immune regulatory induction process.^16^ Investigators found that stronger immune suppression can be induced by high-dose, oligomerized, linear, and soluble epitope peptides.^369–371^ The change of protein structure and modification of certain amino acids in the peptide can induce immune suppression for the treatment of autoimmune diseases more efficiently.^372^ Early researchers have demonstrated that MBP coupled with diazotized arsanilic and sulfanilic acid (Ars-Sulf-MBP) as well as modification of arginine, lysine, and tryptophan residues of MBP selectively can suppress EAE development.^373,374^ MBP modified by bromide was shown to be effective for EAE treatment.^375^ Furthermore, researchers mixed MBP and hapten for EAE suppression.^376^ Recently, our group designed novel fusion proteins to treat EAE and revealed related mechanisms about how cognate antigens suppress CNS inflammation and EAE progression.^377^

The route of administration is particularly important to achieve better immune tolerance effects. Oral administration and inhalation of MBP were reported in 1988 and 1993 respectively.^378,379^ It is worth mentioning that drugs can enter the CNS directly through the olfactory nervous and trigeminal nerves, and indirectly through the nasal mucosa by intranasal or inhalational administered for better CNS drug delivery.^380,381^

#### Altered peptide ligands (APL)

**Fig. 7:**
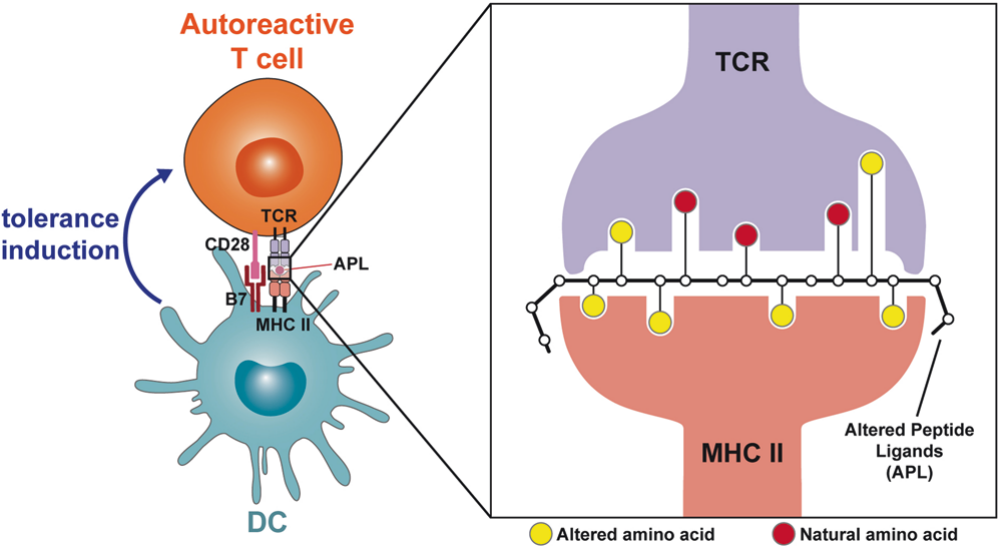
Altered Peptide Ligands (APL) for tolerance induction in TCR-peptides-MHCII. Several amino acid substitutions in key TCR identification positions can cause the signal transmission process obstacles which can affect the immune activation and induce immune tolerance. The yellow circles represent natural amino acids; the red circles represent altered amino acids

Altered peptide ligands (APL) are natural peptide analogs with at least one amino acid substitution at TCR positions (Fig. 6). Different substitutions in particular residues may induce different T cells responses,^382,383^ even though APL possess similar binding between MHC and TCR to natural peptide. Some APL cannot induce a complete signal for T cell proliferation, hence the immune anergy can be induced in this way^384,385^ (Fig. 7). Early researchers have attempted to utilize single amino acid substitution in peptide segments of MBP for the prevention and treatment of EAE.^386–388^ Corresponding APL can also be synthesized by molecular mimicry techniques of microbes to prevent EAE.^389^ Besides, some groups designed the MHC anchor-substituted variant of PLP_139-151_ (145D, HSLGKW**D**GHPDKF) that the seventh amino acid was replaced by aspartic acid and demonstrated the 145D will not induce the acute hypersensitivity reaction.^390^

In recent years, APL is still being used for the treatment of autoimmune diseases. The novel 3aza-MBP APL which contains aza substitutions increased protease resistance property and effectively suppressed EAE disease progression.^391^ Besides, some investigators also demonstrated that MBP_87-99_ (Ala91, Ala96) APL cyclo can promote the bond to HLA-DR4 and induce antigen-specific immune regulation^392^ and others validated cyclic MOG_35-55_ can reduce the pathological process of EAE.^393^

APL of p55-70 of Imogen38 (Imogen38p_55-70_ APL) can inhibit the proliferation of β-cell reactive T-cell clone but fail to induce classical β-cell reactive T cells anergy. In addition, the APL cannot down-regulate TCR/CD3 complexes.^394^ Treatment of NOD mice with IGRP_206-214_ APLs is inefficient for T1D. Thus, it is necessary to test the dose of APL as well as the affinity between APL, MHC and TCR.^395^ In some clinical trials, APL possesses a certain potential for induction of immunosuppression.^396–398^ However, a small portion of the patients show hypersensitivity reactions which lead to disease progression in certain early clinical trials using APL for MS treatment.^399,400^ It was considered that the APL therapy is more appropriate for Th1-mediated autoimmune diseases because APL can promote the shift away from Th1 cytokines to Th2 cytokines and this can be an explanation for hypersensitivity reactions.^400^ Besides, these APLs all are used in RRMS, APLs for other types of MS have not been reported yet.

#### MHC-autoantigen peptides

Naïve T cell activation relies on 3 signals: (1) interaction between TCR and peptide/MHC (signal 1); (2) co-stimulatory molecules (signal 2); (3) cytokines and chemokines (signal 3).^401–405^ Rather than autoantigen being uptake and presented by APCs, soluble peptide/MHC (pMHC) can directly interact with T cells without co-stimulatory signals. Anergy T cells will thus be induced if only the existence of the first signal while the co-stimulator is missing, and it can facilitate further immune suppression or inhibit the avidity maturation of pathogenic T cells.^406,407^

Accordingly, pMHC complexes are applied in autoimmune disease treatment and Sharma et al. reported the first strategy of I-A^s^ protein-MBP_91-103_/ PLP_139-151_ for EAE therapy in 1991.^408^ Studies using MHC II linking acetylcholine receptor α chain (AChRα_100-116_ or AChRα_144-163_) effectively inhibited experimental autoimmune myasthenia gravis (EAMG)^409,410^ and DR2-MOG_35-55_ can suppress EAE development.^411^ The stable complexes composed of two-domain MHC II and MBP_69-89_ can inhibit and detect encephalitogenic T cells.^412^ Subsequently, investigators validated that the I-A^s^/PLP_139-151_ peptide (RTL401) can induce cytokine switch, promote the Th2-related cytokines expression in CNS, and inhibit the encephalitogenic potential of specific pathogenic T cells.^413^ Peptides-MHC II dimer was also designed for T1D and achieved the expected effect.^414,415^ Recently, Urbonaviciute et al. reported that MHC II- galactosylated collagen type II (COL2) can target the antigen-specific TCR via positively charged tags to expand VISTA-positive nonconventional Tregs for RA.^416^

### Biomaterials-based new strategies for autoantigen delivery

The induction of immune tolerance is affected by several factors including antigen dosage, antigen administration route, and delivery system.^417^ Biomaterials facilitate new strategies to induce immune tolerance by providing accurate delivery of autoantigens to the target organs and controlled release of therapeutics.^418–420^

#### Microparticles delivery systems

Nanoparticles have been used for drug delivery and disease treatment, and some nanoparticles have expanded into extensive clinical applications.^421,422^ The size, surface charge, shape, hydrophobicity, and constituent materials co-determine the drug loading efficacy and organs/cell targeting ability.^423,424^ Some nanoparticles themselves have inflammatory inhibitory effects.^425–427^ Nanoparticles have been extensively investigated in autoimmunity disease treatment.^428–430^

Investigators developed a dual peptide nanoparticle platform which delivers antigen peptides for primary signal and other peptides (LABL, binding with ICAM-1) for inhibitory of co-stimulatory signal. The NPs_LABL+MOG_ is designed for EAE treatment by this platform, which is more effective than NPs_MOG_ for the reduction of myelin sheath inflammatory infiltration and induction of immunosuppression.^431^ Polystyrene or biodegradable poly(lactide-co-glycolide) (PLG) microparticles bearing PLP_139-151_ can be taken up by macrophages expressing the MARCO receptor and this process is mediated by Tregs, T cell anergy and the activation of abortive T cell. These microparticles carrying PLP_139-151_ can suppress the autoimmune progress and prevent epitope spreading via apoptotic clearance pathways to inactive pathogenic T cells.^432^ Based on this principle, low-cost, safe and good biodegradable PLG coupled with PLP_139-151_ has also verified that it can reduce a series of inflammatory cells and inhibit the epitope spreading in the relapsing-remitting EAE model.^433^ In another further study, PLG NPs-PLP_139-151_ significantly downregulates the positive co-stimulatory molecules and remains high in negative co-stimulatory molecules.^434,435^ Selective targeting of liver sinusoidal endothelial cells (LSEC) using NPs delivering autoantigen peptides can induce antigen-specific Tregs and protect mice from autoimmune diseases.^436^ Phospholipid phosphatidyl serine-liposomes (PS-lipo) loading Insulin A and B peptides can also induce tolerance APCs and prevent T1D.^437^ Wilson et al. modified the autoantigens by synthetic glycosylation (N-acetylgalactosamine or N-acetylglucosamine) which can target the liver and induce tolerance more easily. Besides, these modified autoantigens can expand the specific Tregs in T1D, MS, and other autoimmune diseases mice models.^438–440^

Some investigators packaged autoantigen into gold nanoparticles (AuNPs) owing to their features of ease of synthesis, ease of shaping, ease of functionalization and facilitating internalization.^441–443^ In addition, AuNPs are validated to have an anti-inflammatory effect by inhibition of leukocyte migration and cytokines secretion which attracts us to use it to treat autoimmune diseases.^444^ Wegmann et al. designed multiple Ag peptides (MAPs) containing eight PLP_139-151_ peptides around dendrimeric branched lysine core for the treatment of relapsing EAE.^445^ Functional amphiphilic hyperbranched (HB) polymers can precisely control molecular weight and chemical composition to achieve good biocompatibility, expected drug metabolism and accurate targeting for drug delivery.^446–448^ Our group has shown that functional amphiphilic HB polymers can efficiently deliver autoantigen and induce immune tolerance by inducing autoreactive T cell deletion^449^ (Fig. 6).

**Fig. 8:**
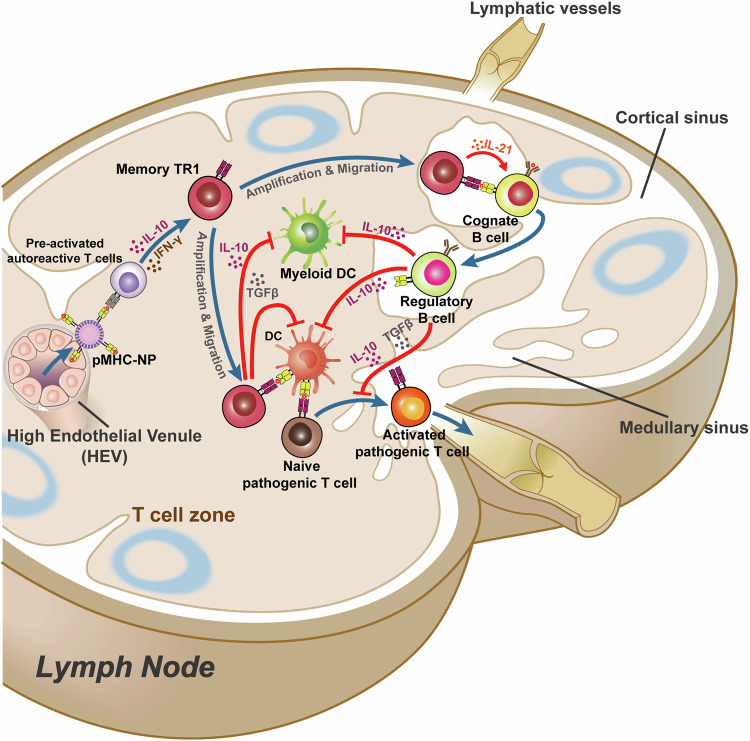
The framework of the establishment of antigen-specific immune regulatory networks by pMHC II-NPs. pMHC II-NPs can be recognized by pathogenic T cells when enter the lymph node through high endothelial venule (HEV) in the T cell zone. Owing to the absence of costimulatory molecules and the action of IL-10, the pathogenic IFN^+^CD4^+^ Th1 will differentiate into memory TR1. The TR1 cells can be amplified and migrate to the specified location before interacting with DCs and cognate B cells. B cells can differentiate into regulatory B cells (Bregs). DCs may dampen the ability of activating pathogenic T cells assisted by relevant anti-inflammatory factors. Meanwhile, the Bregs and TR1 can further regulate the antigen-specific regulatory networks and blunt the autoantigenic and pathogenic cells. The suppression induced by pMHC II-NPs is disease-specific and self-limiting

Researchers also attempted to improve treatment strategies based on pMHC for antigen-specific therapy by nanoparticles in recent years.^450,451^ It has been reported that systemic delivery of nanoparticles coated with pMHC II (pMHC II-NPs) can up-regulate IL-10 and T_R_1-related markers in T_R_1 poised, antigen-experienced CD4^+^ T cells (Fig. 6). The group showed that pMHC II-NPs triggered the expansion of T_R_1 like cells to promote the formation of immune regulatory networks and can restore motor function in EAE mice.^452^ Regulatory B cells are also a potential immune regulatory cells population^124,125^ and play a pivotal role in the antigen-specific regulatory network induced by pMHC II-NPs^452^ (Fig. 8).

By analyzing transcriptional markers, Solé et al. pointed out that the production of FOXP3^-^IL-10^+^ Treg1 cells originates from the Tfh cells via BLIMP1-dependent manner and furthermore confirmed the important role of the pMHC therapy method for autoimmune diseases.^453^ Vacchio et al. reported transcription factor Thpok was necessary for driving Bcl6 and Maf expression to promote differentiation from CD4^+^T cells to Tfh cells.^454^

Targeting IL-2 to induce Tregs for the treatment of autoimmune diseases attracted more attention in recent years.^455,456^ Investigators tried to apply this method to pMHC and then designed tolerogenic microparticles (tol-MPs) loaded with rapamycin (RAPA), biased fusion IL-2 protein and peptide-MHC II tetramers for EAE treatment.^457^ The designed tol-MPs supported Treg expansion and promoted sustained disease reversal of EAE mice.^457^ Umeshappa et al. showed that broad liver autoimmune disease suppression can be induced by T_R_1 cell formation via pMHC II-NPs displaying autoantigen epitopes in an organ rather than disease-specific manner.^458^ For the single-chain pMHC complex (scKd.IGRP) designed by another group, the peptides are covalently attached with β2-microglobulin (β2m) which is linked with MHC I H-2Kd. The group suggested that pMHC I can induce the apoptosis of CTLs.^459^

Tsai et al. utilized NPs to deliver T1D-related peptides-MHC complexes for monospecific resistance of the T1D development, and demonstrated that pMHC can expand the memory-like and autoregulatory CD8^+^ T cells.^460^ NPs coated with pMHC can blunt T1D progression and restore normoglycemia in diabetic animals.^460^

#### Transdermal microneedle patches

**Fig. 9:**
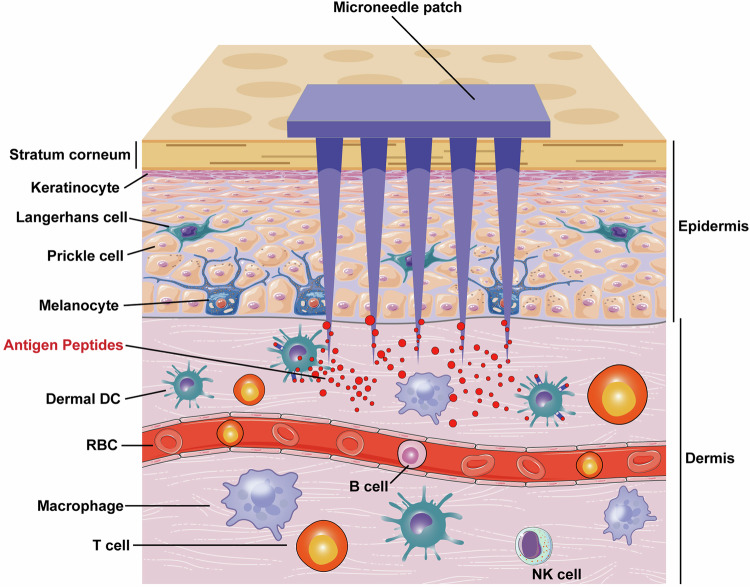
The sketch of antigen peptides delivery by microneedle patch. The microneedle delivery system can deliver antigen peptides to the dermis where there are multiple types of APCs including Langerhans cells, macrophages, and DCs. The abundance of APCs located in the dermis layers makes it an attractive location to deliver antigen peptides for induction of immune tolerance. (Part of the figure was modified from Servier Medical Art(http://smart.servier.com/), licensed under a Creative Common Attribution 4.0 Generic License. (https://creativecommons.org/licenses/by/4.0/)

The skin has abundant APCs and other immune cells (Dermal DCs, Langerhans cells, macrophages, dermal γδ T cells, etc.) which makes it an attractive target for antigen-specific immunotherapy^461–463^ (Fig. 9). Hence, microneedle (MN) administration can effectively promote APCs in the skin to engulf these autoantigens and induce immune regulatory response.^464–466^ Researchers reported a dry-coated MN binding with the topical steroid which can promote longer-retention in the skin. This delivery way can transport autoantigen to the skin for T1D treatment and it promotes the antigen presentation for tolerogenic APCs more strongly than ID injection.^467^

Dul M at el. have employed the MN delivery system, MicronJet600, to target the Langerhans cells in the skin for delivering peptides coupled with gold nanoparticles^468^ (Fig. 6). The addition of gold nanoparticles is validated to expand the distribution of poorly-soluble peptides in lymphoid organs.^469^ MN-gold nanoparticles conjugated with proinsulin peptide (C19-A3 GNP) were designed for T1D treatment.^470^ Another group designed a MN delivery system which includes peptides, diluents, and surfactants, and reported that 86% of therapeutic payload can be delivered to local skin tissue just in 150 s.^471^ A similar study was also reported for RA treatment.^472^

Overall, MN can cause fewer lesions as well as no skin layer distension compared with traditional needles, and furthermore, it can target the APCs in the skin to present the autoantigen peptide efficiently for a longer time with safety and painlessness.^473–476^ Some transdermal patch is currently applied in clinical trials for MS and has shown safety and well toleration.^477^

#### Soluble antigen arrays

Soluble antigen arrays (SAgAs) are new antigen-specific immunotherapies strategies that contain small hyaluronic acid (HA) chains backbone. The peptides can be conjugated onto HA by hydrolysable linkers (hSAgAs) or stable click chemistry linkers (cSAgAs) and delivered to the body via the multivalent, soluble and linear form.^478^ Investigators combined a hybrid insulin peptide and a mimotope as SAgAs and showed efficacy for T1D prevention.^478^ The group also reported that SAgAs can direct the response of epitope-specific T cells.^479^

SAgAs are also validated to induce the desensitization of pathogenic B cell populations and the restoration of the healthy phenotype of autopathogenic APCs in the EAE model.^480,481^ Furthermore, the cSAgAs had a better performance in the antigen presentation process.^478–481^

#### Biomaterials co-delivering autoantigen, immunoregulatory molecules and drugs

Biomaterials loaded with the combination of autoantigen peptides, a series of immune suppression cytokines, and immunosuppressive drugs can inhibit the progression of autoimmune diseases (Fig. 6).

Poly(lactic-co-glycolic acid) (PLGA) and poly(lactic acid) (PLA) have good biocompatibility, immunosuppressive drug loading capacity and appropriate size for application in tolerogenic vaccination. Some researchers demonstrated that PLGA itself can down-regulate the expression of MHCII, CD80 and CD86, and resist DC maturity after lipopolysaccharide (LPS) stimulation which is related with that PLGA can derive lactic acid to inhibit the phosphorylation of TAK1 and then suppress NF-κB activation. Significantly, the immune suppression effect depends on the molecular weight of PLGA, and the higher the molecular weight, the longer the time to immune tolerance induction.^427^ Meanwhile, PLGA can promote the continuous release of antigens and immune regulatory cytokines,^482^ which is beneficial for Treg induction.^483^ Biomaterials based on the PLA system also are potential tools for immune modulation.^484^ Biomaterials combined with immune suppression cytokines and immunosuppressive drugs can further reduce immunogenicity and induce the tolDCs in vivo.^327,482^ Besides, PLGA/PLA-NPs have held approval for many applications in clinical diagnosis and treatment by the FDA.^482,485–487^ Cappellano et al. designed an inverse vaccine containing PLGA NP loaded with MOG_35-55_ and IL-10 for EAE treatment.^488^ Nanoparticles containing PLGA, CD22L, autoantigen glucose-6-phosphate-isomerase (GPI) and RAPA were shown to induce B cell tolerance (measured by the low anti-GPI antibodies and decreased antibody-secreting plasma cells) as well as T cell tolerance (measured by the expansion of Tregs).^489^ In another report, PLGA-NPs-PLP_139-151_ coupled with RAPA inhibited the activation of antigen-specific T cells and B cells, meanwhile induced Tregs and Bregs in SJL mice and protected from EAE development by s.c. or i.v. administration.^490^ Further study demonstrated the robustness of induced tolerance even under antigen rechallenge with TLR7/8 agonist or complete Freund’s adjuvant (CFA) and the transferrable tolerance of antigen-specific Tregs to EAE.^491^

Antigen-specific PLGA dual microparticle (dMP) system which contained two sizes of MPs, one is phagocytosable MPs about 1 μm for antigen delivery and the other is non-phagocytosable about 50 μm for encapsulating factors delivery, was designed for the treatment of mouse model for MS and showed complete protection against disease.^492,493^ A similar study is also reported about acPLG-PLP-TGF-β,^494^ PLGA NPs-MOG/MHC-TGF-β1 coupled with PD-L1 Fc and CD47 fragments^495,496^ and PLG- BDC peptide binding GM-CSF.^497^ In NOD mice, the dMP system induces immature phenotype and LPS-activated resistance phenotype of DC and also prevents the T1D development to a certain extent.^498,499^

Moreover, studies have shown that colloidal gel vaccine containing alginate, chitosan and autoantigen peptide can induce long-term suppression of EAE^500^ (Fig. 6). Park et al. developed a tolerogenic nanovaccine to deliver MOG peptide and dexamethasone loaded on an abatacept-modified polydopamine core nanoparticle (AbaLDPN-MOG). AbaLDPN-MOG can reduce IFN-γ secretion by blocking the interaction between CD80/CD86 and CD28.^501^ NPs-MOG_35-55_ coupled with 2-(1’H-indole-3’-carbonyl)-thiazole-4-carboxylic acid methyl ester (ITE) also induce the FoxP3^+^ Treg differentiation in vitro and inhibit the EAE model.^502^ Yeste A at el. have designed nanoparticles to deliver both a tolerogenic molecule and β-cell antigen proinsulin to induce tolerogenic phenotype in DCs through induction of SOCS2 and suppressed autoimmune diabetes in the nonobese diabetic mice model.^503^ Similarly, the NP-allergen epitope fragment coupled with adjuvant R848 (TLR7 ligand for reducing the allergy symptoms) protected mice from food allergic responses.^504^ At an early stage, Capini et al. also reported that RA-related Ag-NF-κB inhibitor-egg phosphatidylcholine liposomes can induce Ag-specific FoxP3^+^ Tregs and inhibit the clinical symptoms of RA.^505^ Upregulation of PD-L1 is observed in mice treated with calcitriol-antigenic peptide-liposomes, and disease development has been alleviated in the corresponding RA model and vasculitis models.^506^ Besides, Multiepitope citrullinated peptide (Cit-ME)-Rapa-Lipid coating calcium phosphate nanoparticles (LCPs) can inhibit the consistent inflammation in RA models.^507^ A similar formula drug was also validated in another experiment for RA treatment.^508^

Luo et al. did not choose tolerogenic drugs but used CRISPR-Cas9 plasmid (pCas9) combined with antigens and nanoparticles, which can present the antigens and block the CD80, CD86, and CD40 simultaneously. It also promoted the generation and expansion of antigen-specific Tregs.^509^

Thus, co-delivery of autoantigen peptides with other tolerogenic agents is essential to combine multiple signals to induce long-term immune tolerance for antigen-specific immunotherapy.

### Autoantigen coupled probiotics and extracellular vesicles

*Lactococcus lactis* as a versatile and mucosa-targeted vehicle has been applied to carry a series of drugs including peptides in recent years.^510–513^ It has been reported that genetically engineered *Lactococcus lactis* can induce antigen-specific tolerance through oral administration and the utilization of genetically modified *Lactococcus lactis* for celiac disease^514,515^ (Fig. 6). The main advantage of the therapeutic approach is to induce intestinal Tregs (iTregs) differentiation by CD103^+^ DC after the antigen uptake^516^ and facilitate bystander immunosuppression effects by secreting anti-inflammatory cytokines.^517^ The co-delivery of IL-10 and proinsulin via oral administration of *Lactococcus lactis* combined with low-dose anti-CD3 therapy can induce infiltration of autoimmune CD8^+^ T cells and promote the accumulation of Tregs in the pancreas.^518^

Furthermore, extracellular vesicles (EVs) are the cell-natural nanoparticles released from all eucaryotic and procaryotic cells and play a vital role in intercellular communication and material transport, and show great potential in drug or peptide delivery.^519–521^ Meanwhile, EVs contain multiple intracellular proteins and cell surface proteins that are similar to the source cells.^522,523^ These characteristics of EVs are extremely useful for antigen-specific therapy. Oligodendrocyte-derived EVs (OI-EVs) containing multiple myelin peptides naturally have been shown to induce up-regulation of PD-L1 in monocytes as well as IL-10 in immune microenvironment to suppress EAE development^524^ (Fig. 6). Another group designed engineered EVs coupled with HLA-PPI_15-24_ (pre-proinsulin peptide) and PD-L1 to negatively regulate the activation of T cells in T1D.^525^

### Cell-based antigen-specific immunotherapy

#### Chimeric antigen receptor T cells

Chimeric antigen receptor T cells (CAR-T) is a technology biased towards cell engineering by importing a manually designed CAR molecule to the surface of T cells to enable these cells’ efficient stress recognition with targeting cells in the MHC-independent manner.^526–528^ The original intention of this therapy was to achieve precise tumor treatment and it achieved breakthrough results.^529,530^ Due to the precise targeting mechanism, researchers have tried to promote it to other fields such as autoimmune disease treatment^531–534^ (Fig. 6).

The CAR structure is composed of extracellular structures, transmembrane domains and intracellular domains. The extracellular portion is usually a single-chain variable fragment (scFv) and spacer, and the former is connected by heavy and light chain ligands of monoclonal antibodies and can combine the specific antigen.^535^ The transmembrane domains usually come from CD8 or CD28, and these domains are used to connect the extracellular antigen binding domains and intracellular signal transduction domain. The intracellular domains are usually CD28, CD3ζ, and other co-stimulatory molecule domains for T cell activation.^536^

Zhang et al. chose the 4 citrullinated peptide epitopes as the ligands targeting autoreactive B cells to generate engineering T cells. These engineering T cells can kill the hybridoma cells induced by antigenic peptides and the autoreactive B cells from RA patients specifically.^537^ A recent report about a young woman with severe SLE and serious complications accepted the CAR-T targeting CD19 and other B cell epitopes after the failure of treatment with other monoclonal antibodies and glucocorticoids. During the next 7 weeks after treatment, CAR-T cell numbers rapidly increased and the patient did not have any adverse events related with CAR-T.^538^ Zhang et al. also reported the mAb287 CAR (287-CAR) which can target the critical I-A^g7^-B:9- 23(R3) complex for attacking the pathogenic CD4^+^ T cells in NOD mice and demonstrated that these 287-CAR-T can gather in the pancreatic lymph nodes. However, they also reported that a single dose of injection can only delay but cannot prevent the progression of the disease because of the exhaustion of transferred 287-CAR-T.^539^ HLA-DR1 CAR CD8^+^ T cells are designed to target the pathogenic autoreactive CD4^+^ T cells and restrict RA development.^540^ In addition, it has been reported that the engineered CAR-T cells produced by importing mRNA encoding InsB15-23/β2m/CD3-ζ into the CD8^+^ T cell can target the pathogenic CD8^+^ T cells and it offers a new approach to treat T1D diseases.^541^

Ellebrecht et al. designed the chimeric autoantibody receptor (CAAR) expressed human T cells, and CAAR can aim to target the pemphigus vulgaris (PV) autoantigen, desmoglein (Dsg) 3. It has been confirmed that Dsg3 CAAR-T can specifically kill the B cells expressing Dsg3 on BCR, and they can proliferate to prolong the killing effect.^542^ Other groups also designed the NMDA receptor (NMDAR)-CAAR-T which can identify and eliminate the autoantibodies originating from B cell lines in NMDAR encephalitis.^543^

Compared with the engineering of effective T cells, the engineered Tregs have a wider range of applications in autoimmune diseases.^544^ In previous studies, polyclonal and broad-spectrum T cells were also used for autoimmune disease treatment, however, the effectiveness of these polyclonal T cells is not satisfactory.^545–548^ The emerging engineered CAR-Treg can effectively solve this problem. Actually, researchers developed Tregs redirected by antigen-specific chimeric receptor targeting specific antigens and the therapeutic effect has been validated.^549–551^ Fransson et al. tried to engineer the CD4^+^ T cells with CAR targeting MOG in trans with the murine FoxP3 gene which can drive Tregs differentiation and suppress EAE when administered by intranasal cell delivery.^552^ Tenspolde et al. redirected the specificity of T cells to insulin by CAR technology and induced effective T cells to differentiate into Tregs. These CAR Tregs have stable expression, effective inhibition, and long-term existence in NOD mice, but they cannot prevent the disease development in female NOD/Ltj mice significantly.^553^ Other groups also reported that engineering Tregs with anti-InsB_10-23_(InsB-g7 CAR Treg) can down-regulate BDC2.5T effector cells in the pancreas and peripheral lymphoid organs and induce bystander immunosuppression for T1D.^554^

However, CAR-T also has some potential safety hazards including cytokine release syndrome (CRS)^555^ and neurological toxicity.^556^ CRS is the most prevalent adverse effect after CAR-T therapy which can manifest as a strong immune activation and powerful inflammatory storm. Neurological toxicity usually manifests as confusion, myoclonus, and expressive aphasia.^557^ Some researchers found that CAR Treg will change the cellular phenotype with regulatory function and convert to pathogenic autoreactive T cells which is undoubtedly devastating for autoimmune disease patients accepting CAR-T therapy. It is uncertain whether side effects like CRS and neurological toxicity will occur in CAR Treg, but it still needs more attention.^531^

#### Cell engineering beyond CAR-T technology

In addition to the engineering of T cells, other immune cells can be engineered for autoimmune disease treatment. As mentioned above, tolDCs can efficiently induce T cell tolerance and they are also a key target of many therapeutic drugs so that they can be engineered to treat autoimmune diseases^326–328^ (Fig. 6). A group developed the engineering tolDCs by importing lentiviral vectors carrying some specific antigens and IL-10 sequence. These engineering tolDCs can secrete IL-10 and inhibit the autoreactive CD4^+^ and CD8^+^ T cells from celiac disease patients. Besides, these engineering tolDCs can induce antigen-specific Tr1 and prevent the development of T1D in NOD mice.^558^ Gudi et al. engineered DC to express B7.1wa, PD-L1, HVEM-CRD1 or multi-ligand combination which can prevent the CD4^+^ T cells proliferation and related inflammatory cytokines secretion. Researchers use DCs loading mouse thyroglobulin to prevent the development in experimental autoimmune thyroiditis.^559^ In a phase 1b trial, the engineering tolDCs loading with myelin proteins and aquaporin-4 (AQP4) to treat MS patients and induced increase of Tr1 and IL-10 levels successfully without serious adverse events and therapy-related reactions.^560^ VitD3-antigen-specific tolDCs pulsed with MOG_40-55_ ameliorated EAE.^561,562^ OVA-pulsed DCs activated by LPS also alleviated inflammation in OVA-sensitized mice.^563^

Investigators generated MOG mRNA-electroporated tolDCs presenting autoantigen via electroporation with mRNA encoding MOG and demonstrated its capability to stabilize the clinical score in EAE mice.^564^ Besides, engineered bi-specific Tregs expressing TCR cross-reactive to MOG and neurofilament-medium (NF-M) had superior protective properties than engineered Tregs expressing MOG mono-specific TCR.^565^ Other researchers synthesized engineered MBP-specific human Tregs to suppress the development of EAE and demonstrated the induction of bystander suppression.^566^ Qian et al. engineered naïve T cells by importing a retroviral expression system connected with related antigens and verified that these engineered Tregs can exhibit different abilities compared with traditional Tregs.^567^

By cell engineering, the specific antigen can also couple with some other cells for tolerance induction^568–572^ (Fig. 6). Erythrocytes covalently linked to antigenic peptides via the interaction between RBCs endogenous proteins and LPET-sortase covalent intermediate, are designed for protecting against EAE and T1D in an antigen-specific manner.^573^ Peripheral blood mononuclear cells (PBMCs) coupled with 7 myelin-related peptides have been investigated in a phase 1 trial in patients with MS and the results indicated good safety and tolerance of this strategy.^574^ The engineered cells can also originate from the location of inflammation, and Au et al. reported the bioengineering PD-L1 and CD86 functionalized Schwann cells for EAE tolerance treatment.^575^

#### TolDCs cell for antigen-specific therapy

Harry et al. harvested monocytes from RA patients and healthy donors and induced the cells to differentiate into tolDCs using immunosuppressive drugs, immunomodulatory, and vitamin D3 (VitD3). TolDCs established from patients with RA exhibited typical tolerogenic phenotypes and are comparable to those induced from healthy controls.^576^ TolDCs can also be generated from MS patients and T1D patients with the aim of developing therapeutics for these diseases.^577,578^ Recently, VitD3-tolDCs generated from healthy donors and MS patients combined with IFN-β decreased the percentage of activated T cells and induced a shift towards the Th2 profile to inhibit EAE.^579^ These tolDCs derived from the patients themselves may also have a certain antigen-specific inhibitory effect and are safer for transplantation.^576^ Some groups also chose to culture tolDCs derived from patients in vitro with autoantigens which may further increase antigen specificity.^580^ Other groups obtained tolDCs derived from healthy mice and cultured with 2-deoxy glucose (2-DG), and inhibited the experimental autoimmune uveoretinitis (EAU) in vivo^581^ (Fig. 6).

Subsequent studies implicated that the mature induction is required for tolDCs to maintain immune tolerance.^582^ Another study reported that efficient treatment will be achieved only when tolDCs are coupled with disease-related autoantigen peptides.^583^ Boks et al. compared clinical-grade tolDCs generated by coculture with different cytokines (VitD3, IL-10, dexamethasone, TGF-β or RAPA) and demonstrated that clinical-grade IL-10 generated DCs were optimal in tolerance induction.^584^

##### Treg cell therapy

In a 2015 report, the polyclonal Tregs are effective for T1D patients by adoptive transplantation without infusion reactions and related serious adverse events.^585^ The combination of IL-2 and autologous polyclonal Tregs also showed therapeutic effects.^586^ Investigators reported a SLE patient who accepted autologous Tregs enhanced and expanded in vitro can keep the disease stable. These transplanted cells can accumulate in the skin and induce IFN reduction, and enhance Th17-related pathways.^587^ In addition, some researchers directly extracted the MBP-reactive Tregs from Tg4 mice expressing transgenic MBP-reactive TCR and expanded them in vitro. Adoptive transplantation of these cells can improve the EAE condition^588^ (Fig. 6).

### Gene therapy

Gene therapy can overcome the limitation of duplicate injection and some side effects caused by antibodies and cytokines therapy and has enormous potential in autoimmune diseases.^589–591^ We mainly introduce the treatment methods related to nucleic acid vaccines here.

#### DNA vaccine

**Fig. 10:**
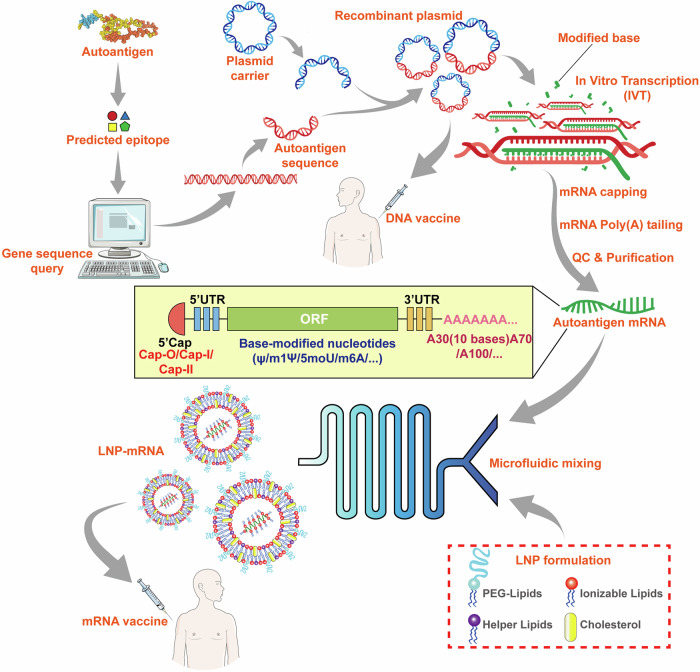
The flowchart of LNP-mRNA and plasmid-DNA vaccines for autoimmune diseases. The encoding of autoantigen is designed by protein and gene databases. DNA sequence fragments encoding the target antigen peptides are inserted into the plasmid vector to synthesize the recombinant plasmids. These plasmids can be used as DNA vaccines after quality control (QC) and purification. Plasmid DNA is transcribed into mRNA by incorporation of the modified bases. Therapeutic mRNA contains 5’cap, 5’UTR, ORF encoding the target protein/peptides, 3’UTR, and Poly(A) tail. Purified mRNA is mixed with LNP (its components are PEG-Lipids, ionizable lipids, helper lipids, and cholesterol) in a Microfluidic mixer to produce the mRNA-LNP vaccines. (Part of the figure was modified from Servier Medical Art(http://smart.servier.com/), licensed under a Creative Common Attribution 4.0 Generic License. (https://creativecommons.org/licenses/by/4.0/)

DNA vaccines have been developed for a long time in numerous medical fields and today’s technologies of DNA vaccine have reached a high level for disease therapy.^592,593^ For autoimmune diseases, DNA vaccine encoding autoantigen/peptides have been used in antigen-specific immunotherapy^594–596^ (Fig. 6). We summarized the preparation process and drawn a flowchart (Fig. 10).

Recently, several strategies have been tested to improve the efficacy of this approach. To target hepatocytes for immune suppression induction, Akbarpour et al. designed ICLV.ET.InsB_9-23_.142T, which consisted of DNA sequence coding antigen peptides, integrase-competent lentiviral vectors (ICLVs), the enhanced transthyretin (ET) hepatocyte-specific promoter, and 142T regulatory elements.^597^ This DNA vaccine showed long-term existence and continuous expression in hepatocytes, and induced specific immune anergy to antigen peptides and bystander suppression to other antigens via Tregs generation.^597^ The adeno-associated virus-related antigen-specific vaccine targeting the liver can also restore the tolerance to EAE.^598^ Some groups also use plant virus nanoparticles such as cowpea mosaic virus and tomato bushy stunt virus to express the p524 in T1D or pLip1 and pFADK2 in RA to treat corresponding autoimmune diseases. They emphasized the peptide scaffold and adjuvant effect of the plant virus nanoparticles and it can provide experience in preclinical testing.^599^

The use of a single β-cell antigen to induce antigen-specific tolerance for the treatment of T1D patients has so far not been successful. Investigators designed DNA vaccines to deliver multi-epitopes from several β-cell antigens. This approach resulted in a broad engagement of antigen-specific CD4^+^ and CD8^+^ diabetogenic T cells and delayed the development of T1D diseases.^600^ In another report, this group showed that DNA vaccines can expand the regulatory CD4^+^ cell phenotype and achieve the best therapy effect via long-term intradermal injection.^601^ In a clinical trial, DNA vaccine encoding proinsulin (BHT-3021) has been shown to reduce the frequency of Proinsulin-reactive CD8^+^ T cells.^602^ However, DNA vaccines encoding GAD glutamic acid decarboxylase (GAD) showed mixed results in NOD mice for the treatment of T1D.^603,604^ More DNA vaccines for the treatment of autoimmune diseases have been put into clinical trials and the efficacy is still uncertain.^605,606^

Besides, DNA vaccines also have the risk of causing insertional mutations because DNA works by entering the nucleus which leads to the exposure to exogenous genes for vaccines.^593^ The DNA vaccines also may promote the generation of anti-DNA IgG autoantibodies, which will lead to the aggravation of autoimmune diseases.^607^

#### mRNA vaccine

In 1961, researchers discovered mRNA and tried to use protamine for mRNA delivery.^608,609^ Unlike DNA vaccines, mRNA-based vaccine has no potential risk to enter the host genome because it does not need to enter the nucleus. However, the characteristics of ease of decay, instability and immunogenicity restrict the application of mRNA to a large extent.^610–612^ Karikó et al. found that naturally occurring modified nucleosides can suppress the immunostimulatory activity of RNA.^613,614^ Furthermore, the modified nucleosides can promote the translational capacity and enhance the biological stability more effectively.^615^ This discovery allowed the fast development of various mRNA-based vaccines and therapeutics. Investigators tried to develop mRNA-based DCs vaccines.^564,616,617^ mRNA can code and produce any protein/peptides and this advantage makes it an ideal strategy to treat diseases that need protein/peptide expression. Moreover, a single mRNA strand can encode several antigens or tandem constructs that contain several epitopes from different antigens (Fig. 6). In 2016, Dastagir et al. have reported the delivery of mRNA with tandem multiple diabetes-associated antigen epitopes by DCs for T1D treatment.^618^

As mentioned earlier, the FDA approved the first liposome complex for small interfering RNA (siRNA) binding for the treatment of a rare disease called hereditary transthyretin-mediated amyloidosis (hATTR) in 2018^294,295^ and it is suggested that liposomes are feasible for transformation in the clinical application of RNA delivery.

Thanks to the great efforts made by several research groups and companies to develop efficient delivery systems and methods to decrease mRNA immunogenicity and improve the transportation efficiency over the past decades, mRNA technology has made a major breakthrough during the COVID-19 pandemic. Pfizer-BioNTech utilized LNPs to prepare the BNT162b2 mRNA vaccine against COVID-19 and achieved great success.^619–621^ LNPs are the most advanced mRNA delivery systems and have shown unique advantages.^622–625^ It is precisely because LNPs promote endosomal escape and thus enhance mRNA translation efficiency.^626,627^ mRNA structure consists of a 5’cap, a 5’ untranslated region (5’UTR), an open reading frame (ORF), a 3’untranslated region (3’UTR), and a poly (A) tail. Each part of mRNA has specific structures and composition to maintain mRNA stability.^628,629^ We summarized the preparation process of the LNP-mRNA vaccines and described the structure of mRNA sequence and the components of LNP (Fig. 10).

Krienke et al. designed nanoparticle formulated 1 methylpseudouridine-modified noninflammatory mRNA (m1Ψ mRNA) vaccine coding autoantigens and tested its efficacy to treat EAE^630^ (Fig. 6). They showed that autoantigen encoding m1Ψ mRNA treatment suppressed disease progress in several mouse models of MS via the expansion of Treg cells and the reduction of effector T cells.^630^ Furthermore, epitope spreading is suppressed via Treg cell-mediated bystander tolerance induced by LPX-m1Ψ mRNA encoding MOG_35-55_.^630^

There is growing interest in designing new LNPs to target different organs and cells for mRNA-based vaccines and therapeutics. Researchers designed a liver-targeting LNP platform to deliver mRNA-encoding allergen epitopes to treat peanut-induced anaphylaxis.^631^ When comparing mRNA delivered by LNPs and mRNA electroporated DCs, LNPs can stimulate T cell responses within a wider antigen-specific T cell subpopulation. Furthermore, nanoparticle-delivered mRNA localized in the spleen preferentially while mRNA electroporated DCs primarily localized in the lung after intravenous injection.^632^ Some researchers designed LNPs containing an anionic phospholipid, phosphatidylserine (PS) to deliver mRNA in the spleen for EAE treatment and achieved a promising efficacy.^633^ Microbubble-assisted focused ultrasound (FUS) technology can increase the BBB permeability for LNP-mRNA and may be more beneficial for mRNA-LNP therapy for MS.^634^

Recent studies reported that EVs extend the function of mRNA-LNPs, protect them from degradation and promote the transport of mRNA between cells.^635,636^ It can improve the efficiency of mRNA transmission as well as the cure rate of autoimmune diseases.

Although the application of mRNA technology is in full swing, we should still pay attention to the future challenges for mRNA development and application in the clinic, which include the delivery of mRNA macromolecules, improvement of the stability of mRNA-delivery carrier and the regulation of mRNA-delivery carrier for immune system.^637^

## Clinical progress of therapeutic drugs

In recent years, we have witnessed the clinical translation of novel therapies for the treatment of autoimmune disorders. Here we overview the FDA-approved drugs and clinical pipelines of antigen-specific immunotherapy for autoimmune diseases. Major breakthroughs have been made in this field, which may pave the way for successful clinical translation of antigen-specific immunotherapies.

### The FDA-approved drugs

Currently, available drugs for autoimmune diseases focus on the targeted blockade of immune inflammation-related membrane surface molecules or cytokines by monoclonal antibody (mAb). Main targets for autoimmune disease treatment include IL-23, IL-17, integrin, TNF, CD20, IL-1, IL-5, IL-6, BAFF/APRIL, etc., and their related receptors or ligands^638^ (Table 1).

**Table 1:** The FDA-approved targeted drugs for autoimmune diseases

Drug	Mechanisms	Administration routes	Indication	Approval time	Research Unit
Target IL-23					
TREMFYA (guselkumab)	Human IgG1λ mAb blocking IL-23 (p19)	s.c.	PsO	2017	JANSSEN BIOTECH (BLA: 761061)
SKYRIZI (risankizumab-rzaa)	Humanized IgG1 mAb blocking IL-23 (p19)	s.c./i.v.	PsO/CD	2019	ABBVIE INC (BLA: 761105)
ILUMYA (tildrakizumab-asmn)	Human IgG1κ mAb blocking IL-23 (p19)	s.c./i.v.	PsO	2018	SUN PHARMA GLOBAL (BLA: 761067)
OMVOH (mirikizumab-mrkz)	Humanized IgG4κ mAb blocking IL-23 (p19)	s.c./i.v.	UC	2023	ELI LILLY AND CO (BLA: 761279)
Target IL-17 and related locus					
COSENTYX (secukinumab)	Human IgG1κ mAb blocking IL-17A	s.c./i.v.	PsO/PsA/AS/ nr-axSpA/HS	2015	NOVARTIS PHARMS CORP (BLA: 125504)
TALTZ (ixekizumab)	Humanized IgG4 mAb blocking IL-17A	s.c.	PsO/PsA/AS/ nr-axSpA	2016	ELI LILLY AND CO (BLA: 125521)
BIMZELX (bimekizumab-bkzx)	Humanized IgG1κ mAb blocking IL-17A/F	s.c.	PsO	2023	UCB INC (BLA: 761151)
SILIQ (brodalumab)	Human IgG2κ mAb blocking IL-17RA	s.c.	PsO	2017	VALEANT LUXEMBOURG (BLA: 761032)
Target Integrins					
TYRUKO (natalizumab-sztn)	Humanized IgG4κ mAb blocking α4β1 and α4β7 integrins	i.v.	MS/CD	2023	SANDOZ INC (BLA: 761322)
TYSABRI (natalizumab)		i.v.	MS/CD	2004	BIOGEN IDEC (BLA: 125104)
ENTYVIO (vedolizumab)	Humanized IgG1 mAb blocking α4β7 interactions with MADCAM-1 and VCAM	s.c./i.v.	UC/CD	2014	TAKEDA PHARMS USA (BLA: 761133)
Target TNF					
AVSOLA (infliximab-axxq)	Humanized IgG1κ mAb blocking TNF-α	i.v.	CD/UC/RA/PsO/PsA	2019	AMGEN INC (BLA: 761086)
INFLECTRA (infliximab-dyyb)		i.v.	CD/UC/RA/AS/PsA/PsO	2016	CELLTRION INC (BLA: 125544)
IXIFI (infliximab-qbtx)		i.v.	CD/UC/RA/AS/PsA/PsO	2017	PFIZER INC (BLA: 761072)
REMICADE (infliximab)		i.v.	CD/UC/RA/AS/ PsA/PsO	1998	CENTOCOR INC (BLA: 103772)
RENFLEXIS (infliximab-abda)		i.v.	CD/UC/RA/AS/ PsA/PsO	2017	SAMSUNG BIOEPIS CO LTD (BLA: 761054)
ZYMFENTRA (infliximab-dyyb)		s.c.	CD/UC	2016	CELLTRION (BLA: 761358)
ENBREL (etanercept)	Humanized IgG1 blocking TNF-α and TNF-β	s.c.	RA/PsA/AS/PsO	1998	IMMUNEX (BLA: 103795)
ERELZI (etanercept-szzs)		s.c.	RA/JIA/PsA/AS/PsO	2016	SANDOZ (BLA: 761042)
ETICOVO (etanercept-ykro)		s.c.	RA/JIA/PsA/AS/PsO	2019	SAMSUNG BIOEPIS CO LTD (BLA: 761066)
ABRILADA (adalimumab-afzb)	Humanized IgG1κ blocking TNF-α interactions with p55 and p75	s.c.	RA/JIA/PsA/AS/CD/UC/PsO/HS/UV	2019	PFIZER INC (BLA: 761118)
AMJEVITA (adalimumab-atto)		s.c.	RA/JIA/PsA/AS/CD/UC/PsO/HS/UV	2016	AMGEN INC (BLA: 761024)
CYLTEZO (adalimumab-adbm)		s.c.	RA/JIA/PsA/AS/CD/UC/PsO/HS/UV	2017	BOEHRINGER INGELHEIM (BLA: 761058)
HADLIMA (adalimumab-bwwd)		s.c.	RA/JIA/PsA/AS/CD/UC/PsO/HS/UV	2019	SAMSUNG BIOEPIS CO LTD (BLA: 761059)
HULIO (adalimumab-fkjp)		s.c.	RA/JIA/PsA/AS/CD/UC/PsO/HS/UV	2020	MYLAN PHARMS INC (BLA: 761154)
HUMIRA (adalimumab)		s.c.	RA/JIA/PsA/AS/CD/UC/PsO/HS/UV	2002	ABBVIE INC (BLA: 125057)
HYRIMOZ (adalimumab-adaz)		s.c.	RA/JIA/PsA/AS/CD/UC/PsO/HS/UV	2018	SANDOZ INC (BLA: 761071)
IDACIO (adalimumab-aacf)		s.c.	RA/JIA/PsA/AS/CD/UC/PsO/HS/UV	2022	FRESENIUS KABI USA (BLA: 761255)
SIMLANDI (adalimumab-ryvk)		s.c.	RA/JIA/PsA/AS/CD/UC/PsO/HS/UV	2024	ALVOTECH USA INC (BLA: 761299)
YUFLYMA (adalimumab-aaty)		s.c.	RA/JIA/PsA/AS/CD/UC/PsO/HS/UV	2023	CELLTRION (BLA: 761219)
YUSIMRY (adalimumab-aqvh)		s.c.	RA/JIA/PsA/AS/CD/UC/PsO/HS/UV	2021	COHERUS BIOSCIENCES INC (BLA: 761216)
SIMPONI (golimumab)	Human IgG1κ mAb blocking TNF-α	s.c.	RA/PsA/AS/UC	2009	CENTOCOR ORTHO BIOTECH INC (BLA: 125289)
SIMPONI ARIA (golimumab)		i.v.	RA/PsA/AS/JIA	2009	JANSSEN BIOTECH (BLA: 125433)
CIMZIA (certolizumab pegol)	Humanized antibody Fab’ fragment blocking TNF-α	s.c.	CD/RA/PsA/AS/ nr-axSpA/PsO	2008	UCB INC (BLA: 125160)
Target CD20					
RIABNI (rituximab-arrx)	chimeric murine/human IgG1κ blocking CD20	i.v.	GPA/MPA	2020	AMGEN INC (BLA: 761140)
RITUXAN (rituximab)		i.v.	RA/GPA/MPA/PV	1997	GENENTECH (BLA: 103705)
RITUXAN HYCELA (rituximab and hyaluronidase human)		s.c.	GPA/MPA/LN (post-marketing experience)	2017	GENENTECH INC (BLA: 761064)
RUXIENCE (rituximab-pvvr)		i.v.	RA/GPA/MPA	2019	PFIZER INC (BLA: 761103)
TRUXIMA (rituximab-abbs)		i.v.	RA/GPA/MPA	2018	CELLTRION INC (BLA: 761088)
ARZERRA/ KESIMPTA (ofatumumab)	Humanized IgG1 mAb blocking CD20 to enhance CDC relative to rituximab	s.c.	MS	2009	NOVARTIS (BLA: 125326)
OCREVUS (ocrelizumab)	Humanized IgG1 mAb blocking CD20 to reduce CDC relative to rituximab	i.v.	MS	2017	GENENTECH INC (BLA: 761053)
GAZYVA (obinutuzumab)	Humanized IgG1 mAb blocking CD20 to enhance ADCC and apoptosis	i.v.	Serum sickness (post-marketing experience)	2013	GENENTECH (BLA: 125486)
Target IL-1 and related locus					
KINERET (anakinra)	recombinant, nonglycosylated form blocking IL-1α	s.c.	CAPS/RA/DIRA	2001	BIOVITRUM AB (BLA: 103950)
ILARIS (canakinumab)	Human IgG1κ mAb blocking IL-1β	s.c.	PFS/Still’s Disease	2009	NOVARTIS PHARMS (BLA: 125319)
ARCALYST (rilonacept)	Dimeric human IL-1R-IL-1RAcP IgG1 fusion protein blocking IL-1	s.c.	CAPS/FCAS/MWS/DIRA/RP	2008	KINIKSA PHARMACEUTICALS (UK), LTD. (BLA: 125249)
Target IL-5 and related locus					
NUCALA (mepolizumab)	Humanized IgG1κ mAb blocking IL-5	s.c.	Asthma/CRSwNP/EGPA/HES	2015	GLAXOSMITHKLINE LLC (BLA: 125526)
CINQAIR (reslizumab)	Humanized IgG4κ mAb blocking IL-5	i.v.	Asthma	2016	TEVA RESPIRATORY LLC (BLA: 761033)
FASENRA (benralizumab)	Humanized IgG1κ mAb blocking IL-5R	s.c.	Asthma	2017	ASTRAZENECA AB (BLA: 761070)
Target IL-6 and related locus					
ACTEMRA (tocilizumab)	Humanized IgG1κ mAb blocking IL-6R	s.c./i.v.	RA/GCA/SSc-ILD/JIA	2010	GENENTECH (BLA: 125276)
TOFIDENCE (tocilizumab-bavi)		i.v.	RA/JIA	2023	BIOGEN MA (BLA: 761354)
TYENNE (tocilizumab-aazg)		s.c./i.v.	RA/JIA/GCA	2024	FRESENIUS KABI USA (BLA: 761275)
KEVZARA (sarilumab)	Human IgG1 mAb blocking IL-6R	s.c.	RA/PMR	2017	SANOFI SYNTHELABO (BLA: 761037)
ENSPRYNG (satralizumab)	Humanized IgG2 mAb blocking IL-6R	s.c.	NMOSD	2020	GENENTECH (BLA: 761149)
SYLVANT (siltuximab)	Humanized IgG1κ mAb blocking IL-6	i.v.	MCD	2014	EUSA PHARMA LIMITED (BLA: 125496)
Target IL-13+/- IL-4					
DUPIXENT (dupilumab)	Human IgG4κ mAb blocking IL-4Rα to inhibit IL-13 and IL-4 signaling	s.c.	AD/Asthma/ CRSwNP/EoE/PN	2017	REGENERON PHARMACEUTICALS (BLA: 761055)
ADBRY (tralokinumab)	Human IgG4 mAb blocking IL-13	s.c.	AD	2021	LEO PHARMA AS (BLA: 761180)
Target IL-12/23					
SELARSDI (ustekinumab-aekn)	Human IgG1κ mAb blocking IL-12 and IL-23	s.c.	PsO/PsA	2024	ALVOTECH USA INC (BLA: 761343)
STELARA (ustekinumab)		s.c./i.v.	PsO/CD/PsA/UC	2009	CENTOCOR ORTHO BIOTECH INC (BLA: 125261)
WEZLANA (ustekinumab-auub)		s.c./i.v.	PsA/Ps/CD/UC	2023	AMGEN INC (BLA: 761285)
Target other loci					
ZINBRYTA (daclizumab)	Humanized IgG1κ mAb blocking IL-2Rα (CD25)	s.c.	MS	2016	BIOGEN (BLA: 761029)
CAMPATH/ LEMTRADA (alemtuzumab)	Humanized IgG1κ mAb blocking CD52	i.v.	MS	2001	GENZYME (BLA: 103948)
ORENCIA (abatacept)	selective costimulation modulator blocking CD80/CD86	s.c./i.v.	RA/JIA/PsA/ aGVHD/	2005	BRISTOL MYERS SQUIBB (BLA: 125118)
XOLAIR (omalizumab)	Humanized IgG1κ mAb blocking free IgE	s.c.	Asthma/IgE-Food Allergy/CSU	2003	GENENTECH (BLA: 103976)
TEZSPIRE (tezepelumab-ekko)	Humanized IgG2λ mAb blocking TSLP	s.c.	Asthma	2021	ASTRAZENECA AB (BLA: 761224)
BENLYSTA (belimumab)	Humanized IgG1λ mAb blocking BAFF	s.c./i.v.	SLE/LN	2011	HUMAN GENOME SCIENCES INC (BLA: 125370)
Antigen mimetic products					
COPAXONE (glatiramer acetate)	Synthetic copolymer based on the structure of myelin basic protein	s.c.	MS	1996	TEVA PHARMS USA (ANDA: 020622)
GLATIRAMER ACETATE (glatiramer acetate)		s.c.	MS	2017	MYLAN (ANDA: 091646)
GLATOPA (glatiramer acetate)		s.c.	MS	2015	SANDOZ (ANDA: 090218)

*AD* atopic dermatitis, *aGVHD* acute graft versus host disease, *CAPs* cryopyrin-associated periodic syndromes, *CD* Crohn’s disease, *CRSwNP* chronic rhinosinusitis with nasal polyposis, *CSU* chronic spontaneous urticaria, *DIRA* deficiency of interleukin-1 receptor antagonist, *EGPA* eosinophilic granulomatosis with polyangiitis, *EoE* eosinophilic esophagitis, *FCAs* familial cold auto-inflammatory syndrome, *GCA* giant cell arteritis, *GPA* granulomatosis with polyangiitis, *HES* hypereosinophilic syndrome, *HS* hidradenitis suppurativa, *JIA* juvenile idiopathic arthritis, *LN* lupus nephritis, *MCD* multicentric Castleman disease, *MPA* microscopic polyangiitis, *MS* multiple sclerosis, *MWS* Muckle-Wells syndrome, *NMOSD* neuromyelitis optica spectrum disorder, *nr-axSpA* non-radiographic axial spondyloarthritis, *PFS* periodic fever syndromes, *PMR* polymyalgia rheumatica, *PN* prurigo nodularis, *PsA* psoriatic arthritis, *PsO* psoriasis, *PV* pemphigus vulgaris, *RA* rheumatoid arthritis, *RP* recurrent pericarditis, *SLE* systemic lupus erythematosus, *SSc-ILD* systemic sclerosis-associated interstitial lung disease, *UC* ulcerative colitis, *UV* uveitis

*ANDA* abbreviated new drug application, *BLA* biologic license application

If there are multiple R&D institutions for a drug, only list one of them

Here we describe several mAb-targeting drugs that have achieved significant clinical treatment effects and some possible side effects.^638^ DUPIXENT (dupilumab) and ADBRY (tralokinumab) which target IL-4/13 can effectively treat atopic dermatitis and asthma. Rituximab targeting CD20, TYRUKO (natalizumab-sztn) and TYSABRI (natalizumab) targeting α4β1 and α4β7 integrins have been found to be efficient in treating MS. Anakinra, canakinumab, rilonacept targeting IL-1 and related loci can be effective for systemic autoinflammatory disease. STELARA (ustekinumab) and WEZLANA (ustekinumab-auub) targeting IL-12/23 (p40) can treat Crohn’s disease effectively. TREMFYA (guselkumab), SKYRIZI (risankizumab-rzaa) and ILUMYA (tildrakizumab-asmn) targeting IL-23 (p19) and COSENTYX (secukinumab), TALTZ (ixekizumab), BIMZELX (bimekizumab-bkzx) and SILIQ (brodalumab) targeting IL-17 or related loci are surprisingly effective for the treatment of plaque psoriasis.^638^

mAbs have some side effects related to their specific targets.^639,640^ For example, serious infections usually occur because of the removal of the target ligand for the mAbs. Patients will experience symptoms including cough, weight loss and low-grade fever. Allergic reaction is another common side effect and this symptom can be very dangerous once it occurs.

In addition, we also emphasize an antigen-mimetic drug, glatiramer acetate, which is a synthetic copolymer, and the component is based on the structure of MBP^641,642^ (Table 1). It has shown astonishing therapeutic effects in animal models and has been applied to clinical MS treatment.^643–645^

### Antigen-specific immunotherapy clinical research progress

Antigen-specific immunotherapy has the high specificity and possesses the potential to induce bystander immune regulation, it holds great potential for the treatment of autoimmune diseases compared with systemic immunosuppressive therapy. There are many different approaches for antigen-specific immunotherapy including whole antigen or peptides, material-based delivery, modified peptides, MHC-peptides, cell-based therapy, and DNA vaccines. Antigen-specific therapies for autoimmune diseases are still in the early stages of clinical application^50,646–648^ and these new approaches hold great promise for successful clinical translation. Here we mainly summarize some related drug designs, progress, outcomes, etc., in clinical trials conducted on antigen-specific immunotherapy (Table 2). Some MS-related clinical trials show hypersensitivity reactions and disease deterioration in individual patients.^399,400,649^ Other clinical trials also show inadequate therapeutic effects.^650–652^ Researchers demonstrated the strong immunogenicity of MBP_83-99_ APL which can induce the cross-reactive with native autoantigen and lead to inflammatory differentiation of naïve T cells. The weak effect of specific treatment occurs not only in MS, but also in T1D.^653–655^ Admittedly, dose and route of administration are the key factors for treatment effects and side effects.^399,653^ However, these conditions are variable and adjustable. The fundamental reason is our limited understanding of the breadth of human autoantigen repertoire and the strategy to deal with the epitope spreading.^358^ For MS, although the role of some myelin has been verified in animal EAE models, the relation with MS is still debatable.^399^ Differences in the epitopes of autoreaction in different patients may provide an explanation for the different treatment effects and side effects in patients while they received the same medicine and routes of administration. Hence, the identification of relevant antigens and personalized autoantigen design need to be addressed urgently for the heterogeneity in individual patients.^358,399^ The combination of autoantigens with immunomodulatory drugs and nanomaterials has made a great progress in animal models,^430,486,656^ however, the toxicity and limitation of nanomaterials and immunomodulatory drugs should also deserve adequate attention in the process of clinical transformation.^657^ Besides, effective biomarkers are urgently essential for the establishment of preclinical diagnosis and long-term monitoring of the disease progress after administration.^358^

**Table 2:** Antigen specific immunotherapy in clinical trials

Name/ID Numbers:	Drug design:	Administration routes:	Disease:	Phase:	Outcomes:	Research Unit:
Whole antigen or peptides						
NCT01536431	Proinsulin (PI) peptide	i.d.	T1D	Phase I/II	Safe and well tolerated, reduction in the population of β-cell-specific effector memory CD8^+^ T cells, high fold change in Foxp3^+^CD25^+^ Tregs, and upregulation IL-10.^672^	Cardiff University
—	MBP (human) + PLP (Bovine)	oral	MS	—	Upregulation of specific TGF-β1 secreting T cells which population may be a distinct subset of T cells (Th3).^361,673^	Brigham and Women’s Hospital and Harvard Medical School
ATX-MS-1467 (NCT01973491)	ATX-MS1 (MBP_30–44_), ATX-MS7 (MBP_83–99_), ATX-MS4 (MBP_131–145_) and ATX-MS6 (MBP_140–154_)	i.d./s.c.	MS	Phase II	Safety is unremarkable, reduction in T1 gadolinium-enhanced (GdE) lesions.^649,674^	Merck KGaA, Darmstadt, Germany
ATX-MS-1467 (NCT01097668)		i.d./s.c.	MS	Phase I		Apitope Technology (Bristol) Ltd
(NCT00223613)	Insulin	intranasal	T1D	Phase III	Failed to prevent the development or delay of T1D for children with HLA susceptibility to diabetes^653^	University of Turku
—	Insulin	oral	T1D	—	Not showing significant effect to prevent the development or delay of T1D.^654^	University of Miami
—	Insulin	s.c./i.v.	T1D	—	Small doses are safe to persons at risk for diabetes but fail to prevent the development or delay of T1D for people at high risk for diabetes.^655^	Massachusetts Medical Society
MBP8298 (NCT00468611)	Synthetic peptide with a sequence corresponding to MBP_82–98_.	i.v.	MS	Phase III	Safe and well tolerated, suppression of anti-MBP autoantibodies in CSF for most patients but not provide significant clinical treatment effect compared with the placebo.^650,651^	BioMS Technology Corp
IMCY-0098 (NCT03272269)	Containing C20-A1 sequence (SLQPLALEGSLQKRG) and proprietary thioreductase motif	s.c.	T1D	Phase I	Safe and well tolerated, not significant decrease in C-peptide was detectable compared with baseline.^675^	Imcyse SA
Peptides-delivery carrier						
NCT00837512	Insulin-Microneedle (depth less than 900 micrometers).	microneedle patch/s.c.	T1D	Phase II/III	Less pain than subcutaneous catheter; faster drug onset time than subcutaneous catheter.^676^	Emory University
NCT01684956	Insulin-Microneedle (MicronJet).	microneedle patch/s.c.	T1D	Phase II	—	Massachusetts General Hospital
—	Skin patch with a mixture of 3 myelin peptides, MBP_85–99_, MOG_35–55_, and PLP_139–155_.	transdermal	MS	Phase II	Safe and well tolerated, activate Langerhans cells and induce unique granular DCs in LN, up-regulate Treg1 secreting IL-10, down-regulate IFN-γ and TGF-β and significant reduction of lesion by MRI.^477,677^	Medical University of Lodz
KAN-101 (NCT04248855)	A liver-targeting glycosylation signature conjugated deaminated gliadin peptides	i.v.	CD	Phase I	Safe and well tolerated, drug is cleared from the systemic circulation in about 6 h.^678^	Anokion SA
ANK-700 (NCT04602390)	A liver-targeting glycosylation signature conjugated MS-related antigens.	i.v.	MS	Phase I	—	Anokion SA
Xemys	CD206-targeted liposomal-MBP_46–62_, MBP_124–139_ and MBP_147–170_.	s.c.	MS	Phase I	Safe and well tolerated, significantly down-regulate MCP-1/CCL2, MIP-1β/CCL4, IL-7, and IL-2, up-regulate TNF-α and promote the normalization of cytokine.^679,680^	Russian Academy of Sciences
Modified peptides						
NBI-5788 (NCT00079495)	APL for modification in the MBP_83–99_ with a replacement Lys at position 91 with Ala.	s.c.	MS	Phase II	No development in disease for patients and no new MRI lesion was detected in 18 months of follow-up, cross-reaction is induced for native peptide but allergy symptoms happened in some patients.^396,400^	Neurocrine Biosciences
CGP77116	APL of MBP_83–99_, sequence modifications in the positions indicated by X (lower case, substitution by a D-amino acid): xXPVVHXFXNIVTPRTP.	s.c.	MS	Phase II	Poorly tolerated and safe, diseases developed and deteriorated in 3 patients and the clinical trial terminated.^399^	National Institute of Neurological Disorders and Stroke, Bethesda, USA
LY3209590	Combination of A-chain, B-chain for APL and IgG2 Fc domain.	s.c.	T1D	Phase II	Safe and well tolerated compared with Insulin Degludec, no significant change for HbA1c from baseline in patients.^681,682^	Eli Lilly and Company
MHC-peptides						
AG284	Solubilized DR2-MBP_84–102_.	i.v.	MS	Phase I	Safe and well tolerated, the frequency of adverse events is similar to placebo, no significant treatment effect, and not establish tolerogenic T cells for MBP.^652^	University of California at San Francisco
NCT00411723	RTL1000(containing the outer two domains of HLA-DR2)-MOG_35-55_.	i.v.	MS	Phase I	Dose of 60 mg or less is safe and well tolerated, significantly and effectively treating relapses MS development.^683^	Artielle ImmunoTherapeutics
Cell-based therapy						
(NCT00445913)	Autologous induced tolDCs in vitro.	i.d.	T1D	Phase I	Safe and well tolerated, no significant difference compared with baseline.^578^	University of Pittsburgh
(NCT01210664)	Autologous induced CD4^+^CD25^high^CD127^-^Tregs in vitro.	i.v.	T1D	Phase I	Safe and well tolerated, the transferred Tregs was long-lived in vivo for patients and increase Treg suppressive activity in vitro.^585,586^	University of California, San Francisco
(NCT01352858)	Autologous induced tolDCs by autologous synovial fluid.	arthroscopical injection	RA	Phase I	Safe and well tolerated but no significant clinical treatment effects were detectable.^684^	Newcastle University
(ISRCTN06128462)	Autologous induced Tregs in vitro.	i.v.	T1D	Phase I	Safe and well tolerated, the transform from naïve CD62L^+^CD45RA^+^ to memory CD62L^+^CD45RA^-^ Tregs and decrease in serum levels of proinflammatory cytokines.^685^	Medical University of Gdansk
—	Autologous CD4^+^ CD25^high^ CD127^-^ Tregs.	i.v.	T1D	Phase I	Safe and well tolerated, significantly high plasma C-peptide levels in treated group.^686^	Medical University of Gdansk
—	TolDCs pulsed with proinsulin peptideC19-A3.	i.d.	T1D	Phase I	Safe and well tolerated, β-cell function and diabetic condition keep a stable level in 6 months of monitoring and all patients with stable HbA1c values.^687^	Leiden University Medical Center
—	RBCs coupled with MBP_13–32_, MBP_83–99_, MBP_111–129_, MBP_146–170_, MOG_1–20_, MOG_35–55_, and PLP_139–154_.	i.v.	MS	Phase I	Safe and well tolerated, reduction in specific T cell for myelin peptides in high dose group, induce generation of IL-10, Tr1 and Tregs.^688^	University of Zurich
NCT02283671	TolDCs coupled with MBP_13–32_, MBP_83–99_, MBP_111–129_, MBP_146–170_, MOG_1–20_, MOG_35–55_, PLP_139–154_ and AQP4_63–76_.	i.v.	MS/NMOSD	Phase I	Safe and well tolerated, induce the generation of Tr1, specific T cells, and PBMCs secreting IL-10 and decrease memory CD8^+^ T cells and NK cells.^560^	Sara Varea
—	PBMCs coupled with MBP_13–32_, MBP_83–99_, MBP_111–129_, MBP_146–170_, MOG_1–20_, MOG_35–55_, and PLP_139–154_.	i.v.	MS	Phase I	Safe and well tolerated, decrease antigen-specific T cells (higher dose) and stabilize the frequency for Tr1, Tregs, and TH2 cells.^574^	Center for Molecular Neurobiology, 20251 Hamburg, Germany
NCT05451212	CAAR-T targeting MuSK.	i.v.	MG	Phase I	—	Cabaletta Bio
DNA vaccine						
BHT-3009 (NCT00382629)	DNA vaccine encoding full-length human MBP.	i.m.	MS	Phase II	Lower 0.5 mg BHT-3009 was safe and well tolerated, reduction in lesion by MRI, autoreactive T cells activity, and anti-myelin autoantibodies in CSF.^605^	Bayhill Therapeutics
BHT-3009 (NCT00103974)	DNA vaccine encoding full-length human MBP.	i.m.	MS	Phase I	Not showing significant effect to prevent the development or delay of T1D.^606^	Bayhill Therapeutics
BHT-3021 (NCT00453375)	DNA vaccine encoding the whole proinsulin molecule.	i.m.	T1D	Phase I	Safe and well tolerated, 1 mg of BHT-3021 is the most effective, good control of HgbA1c and reduction in antigen-specific CD8^+^ T cells.^602^	Bayhill Therapeutics

*MS* multiple sclerosis, *MG* myasthenia gravis, *T1D* type 1 diabetes, *RA* rheumatoid arthritis, *CD* Crohn’s disease, *NMOSD* neuromyelitis optica spectrum disorder, *i.m*. intramuscular injection, *i.v*. intravenous injection, *s.c*. subcutaneous injection. *i.d*. intradermal injection

## Conclusion

This review summarized the epidemiology, mechanisms, and new therapeutic strategies for autoimmune diseases. Continued surveillance of epidemiologic data around the world is needed to improve our understanding of disease risk and disease burden. The development of autoimmune diseases is driven by genetic and environmental factors. With regard to drug development, the treatments of autoimmune diseases have achieved great progress in both antigen-specific immunotherapy and biotherapeutics. The former is still in its infancy in clinical translation, but it has great potential in precise treatment without affecting the whole immune system. Biotherapeutics especially mAbs have successfully applied in clinics for the treatment of autoimmune diseases. The identification of new targets and related biomarkers will enable the development of new biotherapeutics.

Cumulative findings have shown that nanomaterials are promising approaches to deliver autoantigen protein/peptides, DNA, and mRNA for induction of immune tolerance for the treatment of autoimmune diseases.^658–661^ One of the mechanisms behind it is that specific antigens can induce the generation and differentiation of tolerogenic APCs, which will drive the anergy, deletion, and apoptosis of pathogenic CD4^+^/CD8^+^ T cells and induction of Foxp3^+^ Tregs. Furthermore, tolDCs can secrete a series of immunosuppressive cytokines including TGF-β and IL-10 to promote immune anergy.^662^ The additional immunomodulatory molecules/drugs (dexamethasone, ITE, RAPA, etc.) co-delivered by nanomaterials are also a highly efficient approach for antigen-specific therapy by providing multiple suppression-related signals.^663–666^ In addition, these compounds also promote the differentiation of tolDCs and exhibit immunomodulatory effects. Nowadays, many nanomaterials applied in antigen-specific therapy are in the preclinical stage. We believe that the practice of these nanomaterials in clinical trials can further promote the antigen-specific therapy.

The fast development of mRNA-based therapies has attracted people’s attention to many diseases, especially cancer and autoimmune diseases.^637^ Investigators validated that mRNA vaccines have the capacity of inducing Treg cells which execute bystander immunosuppression in animal models for MS.^630^ mRNA technique has several advantages over protein or DNA drugs, including faster manufacturing, lower insertion risk, lower cost and controllable immunogenicity by nucleotide modification. In brief, the mRNA-LNP vaccine has infinite potential in the treatment of many difficult-to-treat diseases, including autoimmune diseases in the future, and now this is just the beginning.^667^ Novel ionizable lipids for mRNA delivery are continuously developed for efficient delivery, better therapeutic effect and safety.^668,669^ Besides, the mRNA-LNP vaccine has been approved for marketing for COVID-19, so it also can provide certain clinical transformation guidance for autoimmune diseases.

The administration routes are also key factors in tolerance induction. For MS, some groups have used the inhalation administration and intranasal delivery strategy for the EAE model and achieved the expected results.^670^ The next research direction can focus on synthesizing drug delivery vehicles for intranasal drug delivery routes. We also emphasize that the effectiveness of drugs varies at different stages of the disease. For MS, almost all drugs only target RRMS.^671^ For example, Tysabri and Fingolimod are approved drugs for RRMS, however, these therapies are ineffective for PMS. Therefore, it is essential to develop new effective therapies for all stages and types of the disease. Besides, the definition of optimal dose conversion, selection of route of administration, and the establishment of effective biomarkers are also huge challenges for individual optimization therapy and disease surveillance in clinical application.

In sum, we emphasized the development and future prospects of highly potential antigen-specific therapies for autoimmune diseases. We summarized antigen-specific therapy including whole antigen protein therapy, antigen modification methods, APL strategies, pMHCs, biomaterials-based delivery methods, tolerogenic cell-based therapy, and gene techniques treatment. Although significant advances have been made in this field, the treatment efficacy of antigen-specific therapy in humans is still uncertain. The development of a tolerable biomaterial delivery system, accurate prediction of specific antigen epitopes, and combination therapy with other immunomodulatory drugs is necessary in both animal research and human clinical trials. Meanwhile, this is also a fundamental challenge we will face in the future. In particular, the decoding of the autoantigen repertoire and epitope prediction can help us better understand the mechanism and origin of autoimmune diseases, and select the corresponding antigen for specific therapy. For the induction of bystander immunity to deal with epitope spreading, researchers must integrate multiple disciplines such as immunology, materials science, biotechnology, bioinformatics, etc. We expect that antigen-specific immunotherapy will soon have clinical application and benefit the patients with various autoimmune diseases.
