# Correction: Evolving understanding of autoimmune mechanisms and new therapeutic strategies of autoimmune disorders

**DOI:** 10.1038/s41392-025-02525-z

**Published:** 2025-12-24

**Authors:** Yi Song, Jian Li, Yuzhang Wu

**Affiliations:** 1 Institute of Immunology, PLA, Third Military Medical University (Army Medical University), Chongqing, China; 2 Chongqing International Institute for Immunology, Chongqing, China

**Keywords:** Immunological disorders, Immunotherapy

Correction to: *Signal Transduction and Targeted Therapy* 10.1038/s41392-024-01952-8, published online 04 October 2024

Since the publication of this review article, the authors received the feedback regarding an inaccurate concept description in the text.^1^ It was previously stated on page 3, column 2, line 46 that “The binding of CD40 on T cells and CD40L on B cells can promote B cells interior recruits the TNFR-associated factors (TRAFs), and reaction molecules include NIK, inhibitor of NF-κB kinase and TPL2 which lead to the activation of transcription factors such as NF-κB and AP1 at last”. Upon checking the related texts in the reference, the authors notice that CD40L is expressed on T cells while CD40 is expressed on B cells. Therefore, the correct text should be “The binding of CD40L on T cells to CD40 on B cells recruits TNFR-associated factors (TRAFs) within B cells. This recruitment activates downstream signaling molecules like NIK, inhibitor of NF-κB kinase, and TPL2, leading to the activation of transcription factors NF-κB and AP-1”.

In addition, Figure 2 should delete “T cells” from the image, because this figure indicates a cellular pattern emphasizing gene regulation downstream of each inflammation-related signaling pathway, rather than just T cells. This figure has been updated below to reflect this change.

In Figure 7, we previously incorrectly reversed the color circle notes in this figure (Correct formulation: the yellow circles represent natural amino acid; the red circles represent altered amino acid) and have now corrected them. This figure has been updated below to reflect this change.

Incorrect Figure 2:
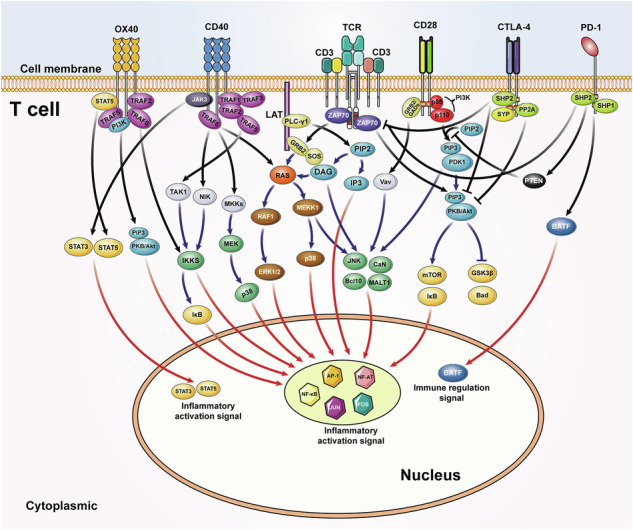


Correct Figure 2:
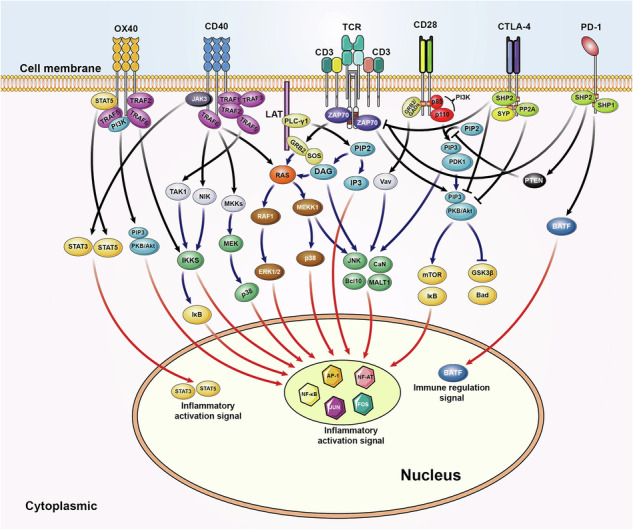


Incorrect Figure 7:
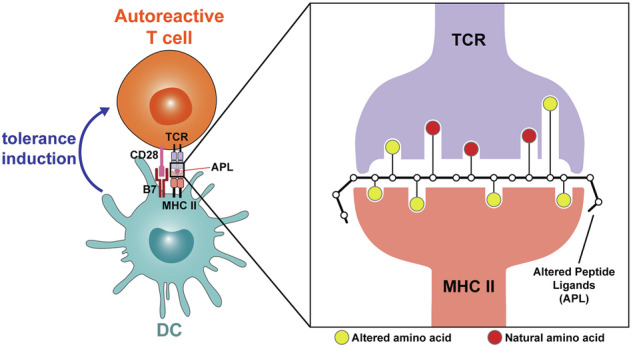


Correct Figure 7:
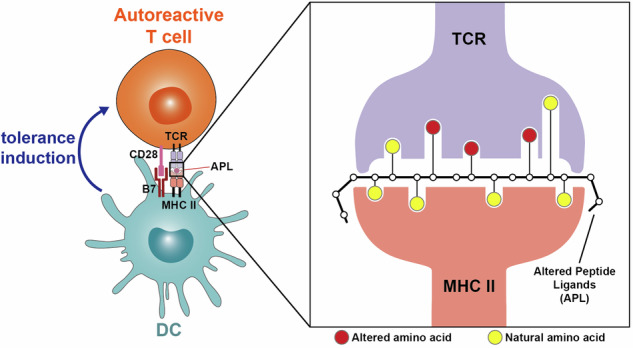


No other findings in this review are affected by these changes.
